# Emotional burden in school as a source of mental health problems associated with ADHD and/or autism: Development and validation of a new co‐produced self‐report measure

**DOI:** 10.1111/jcpp.70003

**Published:** 2025-07-24

**Authors:** Steve Lukito, Susie Chandler, Myrofora Kakoulidou, Kirsty Griffiths, Anna Wyatt, Eloise Funnell, Georgia Pavlopoulou, Sylvan Baker, Daniel Stahl, Edmund Sonuga‐Barke, the RE‐STAR team, Edmund Sonuga‐Barke, Susie Chandler, Andrea Danese, Eloise Funnell, Johnny Downs, Kirsty Griffiths, Myrofora Kakoulidou, Lauren Low, Steve Lukito, Umaya Prasad, Angus Roberts, Emily Simonoff, Daniel Stahl, Anna Wyatt, Georgia Pavlopoulou, Jane Hurry, Sylvan Baker, Graham Moore, Dennis Ougrin, Amanda Roestorf, Rebecca Kirkbride, Claire Lewis, Jordan Altimimi, Beta Balwani, Saskia Barnes, Tiegan Boyens, Zoë Glen, Cj Harris, Charlotte Hillman, Luke Harvey‐Nguyen, Issy Jackson, Amber Johnson, Elisa Ly, Maciej Matejko, Dorian Poulton, Anya Rose, Darren Webb, Archie Wilson

**Affiliations:** ^1^ Department of Child & Adolescent Psychiatry, Institute of Psychiatry, Psychology & Neuroscience King's College London London UK; ^2^ Group for Research in Relationships and Neurodiversity – GRRAND, Research in Clinical, Educational & Health Psychology, Division of Psychology & Language Sciences, Faculty of Brain Sciences University College London London UK; ^3^ Anna Freud National Centre for Children and Families London UK; ^4^ Royal Central School for Speech and Drama London UK; ^5^ Department of Biostatistics and Health Informatics, Institute of Psychiatry, Psychology & Neuroscience King's College London London UK; ^6^ Department of Child and Adolescent Psychiatry Aarhus University Aarhus Denmark; ^7^ Department of Psychology University of Hong Kong Hong Kong China

**Keywords:** Autism, attention‐deficit/hyperactivity disorder, emotional burden, emotion dysregulation, psychometrics

## Abstract

**Background:**

Mental health problems are elevated in adolescents with ADHD and/or autism. Emotion regulation deficits (ERD) have been hypothesised as a key driver of such difficulties. The *Regulating Emotions – Strengthening Adolescent Resilience* (RE‐STAR) programme is examining an alternative pathway from neurodivergence to mental health problems, mediated by elevated *emotional burden* (EB) resulting from the interplay of increased exposure and an unusually intense emotional reaction to commonly upsetting events (CUEs). We present the development and application of the *My Emotions in School Inventory* (MESI), a self‐report questionnaire co‐produced with neurodivergent young people, focusing on EB in schools – a setting thought to be of particular significance in this regard.

**Methods:**

The MESI, containing 25 school‐related CUEs rated on their frequency and the intensity of negative emotions they induce, was completed by secondary school students meeting symptom cut‐offs on clinically validated scales of ADHD (*n* = 100), autism (*n* = 104), ADHD + autism (*n* = 79) and neurotypical students (*n* = 452). Psychometric properties were examined. The ability of the MESI to discriminate adolescents with ADHD and/or autism from neurotypical adolescents, and to predict depression and anxiety, independently of ERD, was explored.

**Results:**

Adolescents in the ADHD and/or autism groups experienced higher CUE frequency and intensity of reaction than their neurotypical peers. Overall levels of EB, most robustly indexed by 24 MESI CUEs, were higher in the three neurodivergent groups, though they did not differ from each other. EB in the autism and ADHD groups was generated by distinctly different CUEs. EB and ERD each contributed independently to the prediction of higher depression or anxiety.

**Conclusions:**

Our findings illustrate the potential value of the MESI as an instrument to measure the contribution of EB alongside ERD in relation to adolescent mental health risks in ADHD and/or autism. Future studies need to investigate its role longitudinally.

## Introduction

Rates of mental health difficulties increase markedly across the adolescent period in the general population (Shore, Toumbourou, Lewis, & Kremer, 2018), creating long‐term risk for mental illness and associated impairment across the lifespan (Katzman, Bilkey, Chokka, Fallu, & Klassen, 2017; Morales‐Muñoz et al., 2023; Schlack, Peerenboom, Neuperdt, Junker, & Beyer, 2021). Such trends are exacerbated in neurodivergent adolescents, such as those with a diagnosis of autism spectrum disorder (ASD; Lai et al., 2019) and attention‐deficit/hyperactivity disorder (ADHD; Danielson et al., 2018). For example, it is estimated that by adulthood around 50% of autistic people (Dow et al., 2021) and 30% of those with ADHD (Wilens, Nierenberg, Rostain, & Spencer, 2008) also have a full clinical depression and/or anxiety diagnosis. The risk of depression is also greater where ADHD and autism cooccur (Factor, Ryan, Farley, Ollendick, & Scarpa, 2017). Furthermore, depression and anxiety can create the context for self‐harm and suicide, which are more common in ADHD and/or autistic than in neurotypical populations (Balazs & Kereszteny, 2017; Blanchard, Chihuri, DiGuiseppi, & Li, 2021). Interventions to improve mental health outcomes for people with ADHD and/or autism are therefore urgently needed. However, our understanding of why neurodivergent adolescents, especially those with ADHD and/or autism, are at particular risk for mental health problems is currently limited.

Recent longitudinal studies have highlighted the role of alterations in emotion‐related traits and implicated mechanisms as mediators of depression risk in adolescents with ADHD and autism (Antony, Pihlajamäki, Speyer, & Murray, 2022; Eyre et al., 2017; Sáez‐Suanes, García‐Villamisar, del Pozo Armentia, & Dattilo, 2020; Seymour et al., 2012; Seymour, Chronis‐Tuscano, Iwamoto, Kurdziel, & Macpherson, 2014). However, interpreting such findings in a way that could be useful for identifying which specific mechanisms to target with interventions is challenging because of the breadth and ambiguity of emotion regulation mechanisms potentially implicated in neurodivergence and/or depression. The *Regulating Emotions – Strengthening Adolescent Resilience* (RE‐STAR) research programme is designed to tease apart the role of two such mechanisms of potential relevance to understanding the ADHD and/or autism‐to‐depression pathway.

First, the hypothesis that depression emerges in adolescents with ADHD and/or autism due to an impaired ability to regulate negative emotions (e.g. anger, frustration, sadness, etc.), caused by neuropsychological dysfunction within the individual has been influential. Indeed, this Emotion Regulation Deficit (ERD) hypothesis is consistent with evidence that: (i) expressions of dysregulated emotion mediate the risk of the emergence of depression in adolescence, in general (Durbin & Shafir, 2008); (ii) young people with ADHD (Shaw, Stringaris, Nigg, & Leibenluft, 2014) and/or autism (McDonald, Cargill, Khawar, & Kang, 2024) display elevated emotional lability, irritability and reactivity, understood as signal phenotypic markers of such dysregulation (e.g. Eyre et al., 2019; Sáez‐Suanes & Álvarez‐Couto, 2022; Seymour et al., 2012, 2014); (iii) the expression of emotion dysregulation in autism and/or ADHD is linked to deficiencies at multiple levels within cognitive systems, including emotion perception (Krause, Linardatos, Fresco, & Moore, 2021; Kret & Ploeger, 2015; Oakley et al., 2022) and awareness (Bunford, Evans, Becker, & Langberg, 2015; Conner et al., 2019; Huggins, Donnan, Cameron, & Williams, 2021; Robert‐Collins et al., 2018); executive control of emotional impulses (Biederman et al., 2012; Seymour et al., 2014); and metacognitive processes (Muniandy, Richdale, Arnold, Trollor, & Lawson, 2023; Pouw, Rieffe, Stockmann, & Gadow, 2013); and (iv) emotion dysregulation‐related traits mediate longitudinal depression risk in autism (Barnes, Ozsivadjian, Baird, Absoud, & Hollocks, 2025; Seymour et al., 2012).

In contrast, the emotional burden (EB) hypothesis, presented here for the first time, shifts the explanatory focus from regulatory deficits *within* the individual to their everyday experiences of common upsetting events and encounters (CUEs), as a source of depression risk; a perspective aligned with a neurodiversity paradigm (Sonuga‐Barke, 2023). This hypothesis comes directly out of the experiences of neurodivergent people as reported in qualitative studies in the early stages of RE‐STAR (Pavlopoulou et al., 2025). These highlighted the frequency with which they experienced CUEs in school as a particularly significant source of emotional challenge and upset (Eccleston, Williams, Knowles, & Soulsby, 2019; Mansfield & Soni, 2024). These qualitative observations were consistent with studies showing that individuals with ADHD and autism experience elevated levels of daily hassles, stressors and negative life events, compared to neurotypical individuals (Eccleston et al., 2019; Taylor & Gotham, 2016) and that certain exposures have heightened salience for them (Beck et al., 2024; Rumball, Brook, Happé, & Karl, 2021). Combining these elements, we operationalised EB as the product of the frequency of CUE experience and the intensity of the induced emotional response to them. We hypothesise that school‐induced EB is an important pathway to depression in neurodivergent people.

Here we report the development of a new self‐report questionnaire for measuring school‐related EB – the *My Emotions in School Inventory* (MESI), which was co‐produced with a neurodivergent *Youth Researcher Panel* (Y‐RP) with autism and/or ADHD (Pavlopoulou et al., 2025; Sonuga‐Barke et al., 2024). It was specifically designed to provide a short and easy‐to‐complete self‐report measure of school‐based EB. It differs from existing self‐report measures of daily stressors (e.g. Byrne, Davenport, & Mazanov, 2007; Compas, Davis, Forsythe, & Wagner, 1987; Weisz et al., 2011) in a number of ways. First, it is a measure focusing particularly on the upsetting emotional experiences, rather than generally positive or negative events (e.g. the Adolescent Perceived Events Scale; Compas et al., 1987). Second, it indexes and integrates the number/frequency of CUEs of different types *and* the degree of emotional upset they generate. In this regard, the MESI is distinct from existing measures of young people's emotional experiences that focus solely on within‐person experiences (e.g. Mazefsky, Yu, & Pilkonis, 2021; Victor & Klonsky, 2016). Third, it developed from the point of view of adolescents with ADHD and/or autism, so it incorporates CUES that they in particular regard as upsetting, alongside other experiences of more general significance. Finally, it focuses on school as a source of EB, and it can easily be implemented by school staff to identify sources of EB that can subsequently be addressed.

Such emphasis on the school setting stems from both our coproduction work and insights drawn from the literature – not only is school a place where young people spend a significant portion of their lives, it is also where neurodivergent young people face some of their greatest emotional challenges (Costley, Emerson, Ropar, & Sheppard, 2021; Pallini, Vecchio, Baiocco, Schneider, & Laghi, 2019), due to social factors such as peer problems or bullying (Maiano, Normand, Salvas, Moullec, & Aime, 2016; Simmons & Antshel, 2021) or conflicts with teachers (MacLean, Krause, & Rogers, 2023), and nonsocial factors such as academic demands or sensory triggers (Costley et al., 2021). The focus also reflected our qualitative research findings that highlighted school as a context where emotional provocations to neurodivergent adolescents were common and intense (Pavlopoulou et al., 2025).

In line with our aim of using the MESI to help identify individuals with heightened EB who are potentially at risk for developing mental health problems, we use data from 735 adolescent mainstream secondary school students: first, to investigate the importance of CUE frequency and the intensity of emotional responses they induce in the conceptualisation of EB as a predictor of mental health problems; second, we calculated EB from the MESI items, scored as the *EB index* (CUE frequency × intensity of emotional reaction) and assessed its psychometric properties; third, to examine the clinical utility of the EB index and its item indicators for distinguishing ADHD and/or autistic adolescent groups from neurotypical peers, and for predicting mental health problems alongside or instead of a measure of intrinsic ERD.

## Methods

### Participants

A total of 746 adolescents aged 11–16 years, were recruited through local NHS clinics, schools, as well as national autism and ADHD charities. Included participants attended mainstream secondary school and had sufficient use of English. Two hundred and thirteen had a verified diagnosis of either ADHD (*n* = 89), autism (*n* = 66) or both (*n* = 58). For the purposes of the current paper, participants were assigned to clinical groups based on these clinical diagnoses (i.e. ADHD and/or autism) or scores above clinical cut‐offs for ADHD and/or autism spectrum disorder on the Swanson, Nolan and Pelham Rating Scale (SNAP‐IV; Swanson et al., 2001; Williams, 2014) and the Social Communication Questionnaire (SCQ; Rutter, 2003), respectively. Those not meeting the cut‐offs on these measures were part of the nonautistic/ADHD group, hereafter referred to as the ‘neurotypical’ group.

### Measures

#### Demographics

Parents/carers provided information about their child's sex, age, diagnosis and ethnicity.

#### School‐based CUEs and emotional burden

Coproduction steps for the MESI development are presented in Figure S1. The MESI was developed based on accounts of everyday emotional experiences of 57 young neurodivergent pupils with diagnoses of ADHD (*n* = 24), autism (*n* = 21) or ADHD + autism (*n* = 12), aged 11–16 years, using a semi‐structured schedule codeveloped with RE‐STAR Youth Researcher Panel (Sonuga‐Barke et al., 2024). Qualitative analysis identified putative CUEs encompassing themes relevant to both autism and ADHD, including (1) social dislocation, alienation and conflict; (2) the need to mask; (3) self‐doubt, loathing, embarrassment and (4) overstimulation/sensory mismatch, which was associated with a range of negative feelings such as anger, shame, confusion (Pavlopoulou et al.,  2025). Academic researchers and the Youth Researcher Panel generated items based on these themes and a prototype 17‐item MESI was created on which participants rated: (a) the *frequency* of the upsetting event, (b) the *likelihood* it would upset them and (c) *how much* (i.e. the *intensity* with which) it would upset them; each rated on a 9‐point scale. This prototype was piloted with 50 adolescents with ADHD and/or autism diagnoses (mean age = 13.4 years, 44% female; Table S1), 42 (84%) of whom were part of the interview study (Pavlopoulou et al.,  2025), though none participated in the main psychometric study. Based on their and the Youth Researcher Panel members' feedback, items were dropped, reworded and added (especially relating to school), and the response format was simplified. The likelihood scale was dropped due to its high correlation with the *intensity* scale (mean Pearson's *r* = .93). The intensity scale was retained in keeping with the Youth Researcher Panel members' preference for measuring the ‘upsettingness’ of the CUE. The final MESI consisted of 25 CUEs (Table S2) rated on 5‐point scales on (a) frequency of upsetting events, referred from here onward as ‘frequency’ (0 = *never*, 1 = *rarely*, 2 = *sometimes*, 3 = *often*, 4 = *frequently*) and (b) how much upset each event would cause, referred here onward as ‘intensity’ variables (0 = *not at all*, 1 = *a little*, 2 = *somewhat*, 3 = *a lot*, to 4 = *extremely*). These variables were used to develop the EB index.

#### ADHD traits

Parents/carers completed the 26‐item SNAP‐IV (Swanson et al., 2001; Williams, 2014) as a measure of their child's ADHD symptoms. Overall scores were calculated by summing and then averaging scores for each of the three symptom clusters: inattentive, hyperactive and inattentive/hyperactive combined, with higher scores representing more ADHD symptoms. Recommended clinical cut‐offs (Bussing et al., 2008) for inattention (1.78), hyperactivity (1.44) and combined (1.67) symptoms were used for assigning participants to the ADHD group.

#### Autistic traits

The SCQ‐current (Rutter, 2003) was completed by parents/carers. The 39 items were scored 0 or 1, with higher scores representing more autistic symptoms. The recommended total score cut‐off of 15 was used for assigning participants to the autism group.

#### Emotion regulation deficits (ERD)

Young people completed the 18‐item short form of the DERS (Victor & Klonsky, 2016), while parents/carers completed the parent version (DERS‐P) (Bunford et al., 2020). Participants rated statements such as ‘When I'm upset, I become out of control’ about themselves, or in the parent/carer's case, about their child, on a 5‐point frequency scale (1 = *almost ever*, 2 = *sometimes*, 3 = *about half the time*, 4 = *most of the time*, 5 = *almost always*). Higher total scores indicated greater ERD. A subset of the parent/carer also completed the 13‐item version of the Emotion Dysregulation Index (EDI; Mazefsky et al., 2021), a measure of emotion dysregulation developed specifically for use in autism.

#### Depression and anxiety symptoms

Young people completed the Patient Health Questionnaire (PHQ‐9; Kroenke, Spitzer, & Williams, 2001) and Generalised Anxiety Disorder Scale (GAD‐7; Williams, 2014), dimensional measures of depression and anxiety symptoms, respectively. Both measures have been validated in adolescents (Fonseca‐Pedrero et al., 2023; Mossman et al., 2017) and were indicators of mental health problems in this study. The PHQ‐9 includes nine items (e.g. ‘Feeling down, depressed or hopeless’) but the ninth item on self‐harm was dropped in this study for ethical reasons. The GAD‐7 includes seven items (e.g. ‘Feeling nervous, anxious or on edge’). In both measures, these items were rated in terms of frequency in the last 2 weeks (0 = *not at all*, 1 = *several days*, 2 = *more than half the days*, 3 = *nearly every day*). Higher total scores in these measures indicated greater mental health problems.

#### Alexithymia

The Toronto Alexithymia Scale (TAS; Bagby, Parker, & Taylor, 1994) was completed by the young people as a measure of difficulties identifying and describing emotions. The questionnaire contains 20 items (e.g. ‘I find it difficult to say how I feel inside’) rated as 0 = *not true*, 1 = *a bit true* and 2 = *true*. Higher scores indicated more difficulties in identifying and describing emotions.

### Procedure

The study was approved by the NHS Health and Social Care Research Ethics Committee (HSC REC A; Ref 23/NI/230047). Eligible families were emailed information about the study and a link to the online consent form. On completion of parental consent and young person consent/assent, parents and young people completed the questionnaires online on a Qualtrics^XM^ platform (Provo, UT). A subsample of young people repeated the MESI after 2 weeks to enable the test–retest reliability examination.

### Sample size estimations

For the measure development, a minimum sample size of *n* = 600 was estimated for exploratory factor analysis (EFA) involving up to 60 MESI frequency and intensity variables (i.e. 30 CUEs). Full consideration of the sample size is described in Appendix S1.4. Briefly, the sample size corresponded with the rule of thumb of 10 respondents per variable (Nunnally, 1978, p. 421), since no a priori measurement model was strongly assumed. For the test–retest reliability examination, a sample size of *n* = 52, allowing for a 10% drop‐off, was estimated for assessing absolute test–retest agreement by intraclass correlation (ICC) = .70 with a 95% confidence interval around the reliability of 15% (Arifin, 2018).

### Statistical analysis

Statistical analyses were completed in R 4.1.1 on RStudio 2023.12.1 using packages *Psych* (Revelle, 2024), *Lavaan* (Rosseel, 2012), *MASS* (Ripley et al., 2024) and *IRR* (Gamer, Lemon, Fellows, & Singh, 2022) and consisted of preliminary analyses, scale development and examination of the convergent validity of the EB construct.

#### Preliminary analysis

Initial item analysis was conducted to establish whether the 50 individual CUE frequency and intensity items: (a) had sufficient variation, that is *not* rated the same way by >80% of participants, (b) demonstrated no signs of separate distributions, for example bimodal across subgroups of neurotypical or neurodivergent adolescents, (c) were not pairwise‐intercorrelated above *r* > .8 and had at least one correlation with *r* > .3 with other items (see Streiner & Norman, 2008) and (d) had less than 5% missing responses. The MESI CUEs were considered for removal if the above criteria were unmet by the frequency and/or intensity variables. We also examined group differences in the participant characteristics and analysed the association between separate total CUE frequency and intensity scores and scores of ERD and mental health measures using correlations and regression analyses.

#### Scale development

The latent factor EB was indicated by the multiplication of a CUE frequency and its intensity (Figure S2), which was a favoured approach for combining data since it preserves rating order (Ajzen & Fishbein, 2008; Amon, Annand, & Holden, 2022; Tofallis, 2014) and ensures zero burden when no CUE occurs or when an individual feels no upset despite experiencing frequent CUEs.

To obtain a robust and parsimonious model of the EB factor, the factor structure of EB was first assessed using exploratory factor analysis (EFA) with ordinary least squares (OLS), applying oblique *Oblimin* rotation to anticipate correlated factors. Scree plots (Cattell, 1966) and parallel analyses (Horn, 1965; Pearson, Mundfrom, & Piccone, 2013) helped identify the optimal factor number. EB indicators were considered for removal if there were factors with fewer than five indicators unless their loadings were >0.7 (Costello & Osborne, 2005). CUEs of which EB indicators had (a) factor loading less than 0.4 or (b) those cross‐loading on two or more factors with secondary factor loading(s) of at least 0.3 or a loading difference of less than 0.2 (Streiner & Norman, 2008) were also considered for removal. Model fit was examined using goodness‐of‐fit criteria including likelihood ratio chi‐square, Tucker‐Lewis Indices (TLI; good fit ≥ .95; Bentler & Bonett, 1980), and the Bayesian information criterion (BIC) for non‐nested models (Gideon, 1978) and model robustness was examined through bootstrapping (Goretzko & Bühner, 2022). The internal consistency of the EB factor was then examined using Cronbach's *α* and item‐total correlation. In this stage, the EB indicator was considered for removal if the Cronbach's *α* increased upon the temporary dropping of an EB indicator or the indicator correlated with the total score <.3, indicating a small contribution of the item to EB measure (Streiner & Norman, 2008).

We then defined the *EB index* as the sum of the frequency × intensity of the remaining CUEs and examined its reliability as a proxy of the EB latent factor (Appendix S1). The 2‐week test–retest reliability of the EB index was examined using ICC for absolute agreement (Streiner & Norman, 2008). Finally, as an additional robustness examination, we examined the measurement equivalence of the final EB latent factor in a series of multigroup confirmatory factor analyses (MGCFA) across the ADHD, autism and combined groups, sex and race (white vs. nonwhite). The broad reconfiguring of the ethnicity to race (white vs. nonwhite) was done due to the relatively small number of participants in some ethnic subgroups (Appendix S1.5).

#### EB index validity

We investigated the validity of the EB index in three ways. First, we compared participant groups using an analysis of variance (ANOVA), applying *post hoc* Tukey's honestly significant difference (HSD) multiple comparison correction. Second, we conducted a series of regression models to investigate the associations among EB index, ADHD/autism group membership, ERD and mental health problems and to examine if EB was associated with mental health problems independently of ERD. We initially regressed EB index, ADHD/autistic traits or ERD separately on depression/anxiety as a dependent variable. Then, we entered EB index and ERD simultaneously as predictors of these mental health problems, controlling for sex, ADHD/autistic traits and alexithymia scores. We added alexithymia (TAS) scores as a covariate in selected analyses to ensure that any differences between ADHD, autistic and neurotypical individuals with regard to EB and/or ERD were not due to more basic differences in emotion understanding and identification, associated with alexithymia, known to commonly occur in autism. Third, we examined whether subsets of CUEs were associated with ADHD and autism group memberships using linear discriminant analyses (LDA) (Appendix S1 for details), applying the threshold of linear discriminant coefficient absolute value of .30 to identify influential CUEs for specific group memberships (Dhamnetiya, Goel, Jha, Shalini, & Bhattacharyya, 2022; Lambert & Durand, 1975). To account for group differences in sex and ethnicity, we conducted sensitivity analyses, adjusting for sex and also race (white vs. nonwhite). The reconfiguring of ethnicity to race differences was again conducted due to the limited number of participants in some ethnic groups.

## Results

### Preliminary analyses

All 50 frequency and intensity variables on the MESI fulfilled the criteria set in the initial item analysis; therefore, no CUEs were removed at this stage (Table S3). Missing MESI frequency or intensity variables occurred in 11 (1.5%) participants, a level considered ‘inconsequential’ (Schafer, 1999). Thus, these cases were excluded, leading to a total of 735 cases in subsequent analyses with groups meeting inclusion criteria for ADHD (*n* = 100), autism (*n* = 104), ADHD + autism (*n* = 79) and those who were neurotypical (*n* = 452); (mean age 13.3, SD 1.32 years; 36% female, 52.7% white) (Table 1). The groups differed in sex (*χ* ^2^[3] = 20.5, *p* < .001), with more females in the autism than the ADHD or neurotypical group; and in ethnic distribution (*χ* ^2^[12] = 105.3, *p* < .001), with higher rates of white and Asian/Asian British ethnicities in the neurotypical than in the ADHD and/or autism groups. Age did not differ across groups.

**Table 1:** Characteristics of included participants

Variables	Overall		ADHD		Autism		ADHD + Autism		Neurotypical		Group statistics			Post hoc
	(*N* = 735)		(*N* = 100)		(*N* = 104)		(*N* = 79)		(*N* = 452)		*χ* ^2^/*F*	df	*p*‐Value	
Age, years (*M*, *SD*)	13.3	1.32	13	1.3	14	1.5	13	1.4	13	1.3	2.6	(3, 731)	*ns*	–
Sex, female (*n* (%))	264	(36)	30	(30)	57	(55)	31	(39)	146	(32)	20.5	3	<.001	Autism > neurotypical***; autism > ADHD***
Ethnicity (*n* (%))														
Asian/Asian British	133	(18.1)	1	(1)	4	(4)	3	(4)	125	(28)	105.3	12	<.001	
Black/African/Caribbean	105	(14.3)	7	(7)	18	(17)	5	(6)	75	(17)				
Mixed	89	(12.1)	13	(13)	14	(13)	13	(16)	49	(11)				
White	387	(52.7)	77	(77)	67	(64)	57	(72)	186	(41)				
Other ethnic group	20	(2.7)	2	(2)	1	(1)	1	(1)	16	(4)				
SCQ (*M*, *SD*)														
SCQ total score	11.2	6.9	11	5.8	17	5.2	19	6.4	8.4	5.4	137.8	(3, 728)	<.001	ADHD, autism, ADHD + autism > neurotypical***; autism, ADHD + autism > ADHD***
DSM‐5 SCI score	7.8	5.0	7.4	4	11	4.5	12	4.6	6.4	4.6	54.2	(3, 714)	<.001	ADHD, ADHD + autism > neurotypical***; ADHD, ADHD + autism > ADHD***
DSM‐5 RRB score	2.6	3.1	3.2	2.9	5.3	2.8	6.2	3.1	1.3	2.2	141.8	(3, 724)	<.001	ADHD, autism, ADHD + autism > neurotypical***; autism, ADHD + autism > ADHD***
SNAP‐IV (*M*, *SD*)														
Combined score	1.1	0.87	2.2	0.51	1.4	0.57	2.2	0.57	0.57	0.53	342.7	(3, 731)	<.001	ADHD, autism, ADHD + autism > neurotypical***; ADHD, ADHD + autism > autism***
Inattention score	1.3	0.96	2.4	0.52	1.7	0.70	2.4	0.56	0.70	0.63	285.7	(3, 731)	<.001	ADHD, autism, ADHD + autism > neurotypical***; ADHD, ADHD + autism > autism***
Hyperactivity score	0.9	0.86	2.0	0.65	1.0	0.64	1.9	0.68	0.44	0.53	393.0	(3, 731)	<.001	ADHD, autism, ADHD + autism > neurotypical***; ADHD, ADHD + autism > autism***
DERS (*M*, *SD*) parent‐rated														
DERS total score	43.0	15.6	52	12	52	13	60	13	36	13	131.1	(3, 720)	<.001	ADHD, autism, ADHD + autism > neurotypical***; ADHD + autism > ADHD, autism***
Lack of emotional awareness	8.7	3.2	9.7	2.8	9.7	2.9	10	2.8	8.1	3.2	17.9	(3, 724)	<.001	ADHD, autism, ADHD + autism > neurotypical***
Lack of emotional clarity	6.5	3.0	7.5	2.6	8.1	2.8	9.2	3.0	5.5	2.6	63.8	(3, 728)	<.001	ADHD, autism, ADHD + autism > neurotypical***; ADHD + autism > ADHD***; ADHD + autism > autism*
Difficulty engaging in goal‐directed behaviour	9.0	4.1	12	3.1	11	3.5	13	2.6	7.1	3.5	118.3	(3, 726)	<.001	ADHD, autism, ADHD + autism > neurotypical***; ADHD + autism > autism**
Impulse control difficulties	6.9	4.1	9.7	3.8	8.3	3.9	12	3.7	5.1	3.0	121.5	(3, 727)	<.001	ADHD, autism, ADHD + autism > neurotypical***; ADHD + autism > autism, ADHD***; ADHD>autism*
Nonacceptance of emotional responses	5.9	3.1	6.1	2.9	7.2	3.2	7.6	3.6	5.2	2.8	24.7	(3, 727)	<.001	Autism, ADHD + autism > neurotypical***; ADHD > neurotypical*; ADHD + autism>ADHD**; autism>ADHD*
Limited access to emotion regulation strategies	6.0	3.4	7.0	3.2	7.5	3.4	8.9	4.0	4.9	2.7	54.0	(3, 727)	<.001	ADHD, autism, ADHD + autism > neurotypical***; ADHD + autism > ADHD***
EDI total score (*M*, *SD*)	30.3	13.1	36	12	33	11	42	14	23	11	16.9	(3, 155)	<.001	ADHD, autism, ADHD + autism > neurotypical***; ADHD + autism > autism*
DERS (*M*, *SD*) young person‐rated														
DERS total score	45.8	15.4	53	14	52	13	57	15	41	14	47.6	(3, 708)	<.001	ADHD, autism, ADHD + autism > neurotypical***
Lack of emotional awareness	9.4	3.1	9.8	2.6	9.7	3.2	11	2.7	8.9	3.2	9.9	(3, 717)	<.001	ADHD + autism > neurotypical***
Lack of emotional clarity	6.9	3.3	7.8	3.1	8.1	3.2	8.7	3.6	6.1	3.1	25.9	(3, 722)	<.001	ADHD, autism, ADHD + autism > neurotypical***
Difficulty engaging in goal‐directed behaviour	9.3	3.9	11	3.2	11	3.4	11	3.5	8.2	3.7	40.5	(3, 719)	<.001	ADHD, autism, ADHD + autism > neurotypical***
Impulse control difficulties	7.0	3.8	9.4	3.9	7.8	3.5	9.9	4.0	5.8	3.2	52.9	(3, 719)	<.001	ADHD, autism, ADHD + autism > neurotypical***; ADHD + autism > autism***
Nonacceptance of emotional responses	6.5	3.5	7.5	3.6	7.3	3.6	7.4	3.8	6.0	3.2	9.7	(3, 718)	<.001	ADHD > neurotypical***; autism, ADHD + autism > neurotypical**
Limited access to emotion regulation strategies	6.7	3.4	7.5	3.4	8.0	3.2	8.3	3.6	5.9	3.2	22.3	(3, 717)	<.001	ADHD, autism, ADHD + autism > neurotypical***
PHQ‐8 total score	6.9	5.8	8.7	5.3	9.3	5.5	9.5	6.1	5.5	5.4	26.1	(3, 725)	<.001	ADHD, autism, ADHD + autism > neurotypical***
GAD‐7 total score	6.6	5.7	8.6	5.4	9.3	5.6	9.4	6.2	5.1	5.2	32.0	(3, 726)	<.001	ADHD, autism, ADHD + autism > neurotypical***
TAS														
TAS total score	17.2	7.6	20	7.0	20	7.1	22	7.3	15	7.1	37.6	(3, 724)	<.001	ADHD, autism, ADHD + autism > neurotypical***
DDF subscale	4.8	2.9	5.5	2.8	5.9	2.6	6.2	2.9	4.1	2.8	21.6	(3, 724)	<.001	ADHD, autism, ADHD + autism > neurotypical***
DIF subscale	5.1	4.1	6.8	3.8	7.0	4.0	7.4	4.1	3.9	3.6	40.9	(3, 721)	<.001	ADHD, autism, ADHD + autism > neurotypical***
EOT subscale	7.4	2.6	8.0	2.6	7.3	2.8	8.5	2.5	7.1	2.5	9.3	(3, 718)	<.001	ADHD > NT**; ADHD + autism > neurotypical***; ADHD + autism > autism**
CUEs total frequency	44.2	18.2	56	16	51	16	57	19	38	16	59.6	(3, 731)	<.001	ADHD, autism, ADHD + autism > neurotypical***; ADHD + autism > autism*
CUEs total intensity	46.0	21.5	53	20	59	20	60	20	39	20	50.5	(3, 731)	<.001	ADHD, autism, ADHD + autism > neurotypical***

Characteristics of the included participants are described across groups, with group difference statistics presented. CUEs, commonly upsetting events; DDF, difficulty describing feelings; DERS, Difficulties in Emotion Regulation Scale; DIF, difficulty identifying feelings; EOT, external‐oriented thinking; GAD‐7, Generalised Anxiety Disorder Questionnaire; PHQ‐8, Patient Health Questionnaire; SCQ, Social Communication Questionnaire; SNAP‐IV, Swanson, Nolan and Pelham Rating Scale; TAS, Toronto alexithymia scale.

*p<.05, **p<.01, ***p < .001.

There was a main effect of group on the parent‐rated (*F*[3, 720] = 131.3, *p* < .001) and young person‐rated ERD (*F*[3, 708] = 47.6, *p* < .001); and on the young person‐rated depression (*F*[3, 725] = 26.1, *p* < .001); anxiety (*F*[3, 731] = 32.0, *p* < .001) and alexithymia (*F*[3, 724] = 37.6, *p* < .001). Across these variables, the ADHD and/or autism groups usually did not differ from each other (see few exceptions in Table 1), but they all scored significantly higher than their neurotypical peers (all *p*s < .001). The ADHD and/or autism groups also experienced higher CUE frequency (*F*[3, 731] = 59.6, *p* < .001) and greater intensity (*F*[3, 731] = 50.5, *p* < .001) than the neurotypical group. The mean, 95%CI and mean ranks of the MESI frequency, intensity, frequency × intensity variables are presented in Table S4.

Total CUE frequency and intensity correlated strongly (*r* = .68). They were also correlated positively with ERD (*r*s > .45), depression (*r*s > .52), anxiety (*r*s > .59) and alexithymia traits (*r*s > .52) (all *p*s < .001; Table 2b). The overall CUE frequency and intensity scores each independently (all *p*s < .001) predicted depression (*b*_frequency_ = 0.48, 95% CI [0.40, 0.56]; *b*_intensity_ = 0.18, 95% CI [0.11, 0.26]) or anxiety scores (*b*_frequency_ = 0.39, 95% CI [0.32, 0.47]; *b*_intensity_ = 0.32, 95% CI [0.24, 0.39]).

**Table 2:** Correlations among total CUE frequency/intensity variables, ERD and mental health measures

	CUE intensity	ERD (yp)	ERD (pc)	Depression	Anxiety	Alexithymia
CUE frequency	.683***	.505***	.604***	.610***	.614***	.564***
CUE intensity	–	.449***	.551***	.516***	.590***	.516***
ERD (yp)	–	–	.607***	.454***	.501***	.524***
ERD (pc)	–	–	–	.688***	.701***	.774***
Depression	–	–	–	–	.815***	.623***
Anxiety	–	–	–	–	–	.639***

CUE, commonly upsetting events; ERD, emotion regulation deficits; pc, parents/carers; yp, young person.

*** *p* < .001.

### Exploratory factor analysis

The scree and parallel plots strongly suggested a one‐factor structure for the EB latent factor (Figure S2), with a potential emergence of a second factor, driven by an EB indicator representing the CUE *‘School staff treating you unfairly e.g., by giving an unnecessary detention’*. When we explored a two‐factor solution, the indicator loaded on a second factor with a loading of 0.92 (Table S4). To achieve a robust psychometric scale while also recognising the item's clinical importance, we kept this CUE in the MESI. However, we excluded it from the final EB measurement model and the EB index computation. The factor analysis of EB using the remaining 24 indicators showed robust bootstrapped loadings ranging from 0.53, 95% CI [0.48, 0.59], to 0.77, 95% CI [0.74, 0.81] and indicator variance *h* ^2^ ranging from 0.28 to 0.59 (Table 3). The model explained 44% of the variance in the data with acceptable model fit (CFI = 0.844, TLI = 0.829; RMSEA = 0.089, SRMR = 0.051, 90% CI [0.085, 0.093]; and BIC = 59.5).

**Table 3:** Factor loadings of the EB indicators

MESI CUEs	*B*	Bootstrapped	*h* ^2^
		95% CI	
1. Peers talking behind my back	0.62	[0.57, 0.67]	0.39
2. Unexpected wait in a queue	0.53	[0.48, 0.59]	0.28
3. Teachers tell me off	0.66	[0.61, 0.70]	0.43
4. Schoolmates ignore me	0.69	[0.63, 0.74]	0.47
5. Teachers don't listen	0.69	[0.65, 0.73]	0.47
6. Teachers don't understand	0.77	[0.74, 0.81]	0.59
7. Last‐minute change of plan	0.67	[0.63, 0.71]	0.45
8. Not being able to do tasks	0.65	[0.60, 0.70]	0.42
9. Being in a chaotic classroom	0.68	[0.64, 0.72]	0.47
10. Boring lessons or tasks	0.62	[0.56, 0.66]	0.38
11. Being stopped doing enjoyable things	0.62	[0.57, 0.67]	0.39
12. Experiencing sensory discomfort	0.66	[0.60, 0.71]	0.43
13. Losing and forgetting things	0.58	[0.52, 0.64]	0.34
14. In trouble for losing or forgetting	0.62	[0.56, 0.67]	0.38
15. Being rushed to complete work	0.73	[0.69, 0.77]	0.53
16. Not understanding others	0.73	[0.69, 0.78]	0.53
17. Staff treating me unfairly	NA	NA	NA
18. Peers teasing and bullying	0.58	[0.52, 0.66]	0.34
19. Being told to try harder	0.73	[0.69, 0.77]	0.53
20. Being accused of something I didn't do	0.64	[0.59, 0.69]	0.41
21. Not doing something quite right	0.69	[0.64, 0.74]	0.47
22. Not allowed to do self‐regulation strategies	0.71	[0.66, 0.74]	0.50
23. Being pressured to do well	0.60	[0.54, 0.64]	0.36
24. Having too many options	0.63	[0.57, 0.68]	0.39
25. Being rushed to move on	0.72	[0.68, 0.76]	0.52

Factor loadings (*B*) of 24 EB indicators and their bootstrapped 95% confidence interval (CI) and item variance (*h* ^2^) are presented. The final model of EB excluded MESI CUE 17 from being an indicator for the EB latent factor. NA, not applicable.

The 24 EB indicators had an excellent internal consistency (Cronbach's *α* = .948). No single indicator omission increased the alpha (Table S6). Item‐total correlation values ranged from .51 to 0.75. No indicators were removed based on this analysis. The final EB Index and the extracted EB factor score had an excellent agreement (ICC = .999, *p* < .001, range .998–.999, all *p*s < .001 across the diagnoses and sex and race subgroups). Two‐week test–retest completed by 46 participants (i.e. 90% of 52 invited participants) had a good absolute agreement (ICC = .80, 95% CI [0.67, 0.89]).

The MGCFA findings showed that the configural, metric and scalar fits across the diagnostic groups, sex and race (white vs. nonwhite) indicated a suboptimal but acceptable metric invariance across groups (Table S7).

### Differences in EB index across the diagnostic groups

**Figure 1:**
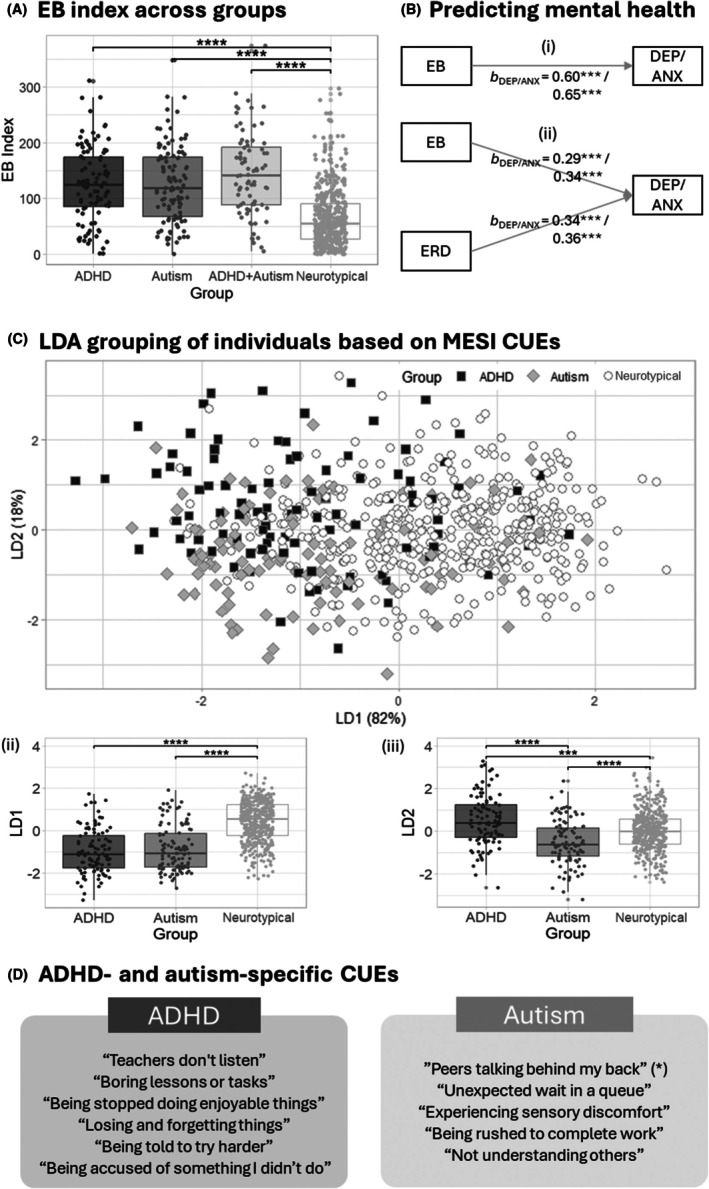
Convergent validity of the emotional burden construct Investigation into the convergence validity of the construct emotional burden found that: (A) EB index was significantly higher in the ADHD and/or autism groups relative to the neurotypical group; (B) EB was strongly associated with mental health (B‐i), and both EB and ERD significantly predicted mental health in the adolescents (B‐ii) in the multiple regression analysis, covarying for ADHD, autistic and alexithymia traits; these findings persisted after adjusting for sex and race differences across groups. (C) The linear discriminant functions (LDs) based on the emotionally burdensome CUEs could predict diagnostic grouping (C‐i), by differentiating particularly, (C‐ii) those with neurodivergence (i.e. autism or ADHD) from the neurotypical groups and (C‐iii) ADHD from autistic adolescents; finally (D) CUEs that are particularly emotionally burdensome to the ADHD group can be differentiated from those burdensome to the autistic group. (*) *‘Peers talking behind my back’* became nondiscriminative when the analysis was adjusted for sex and ethnicity differences across groups. ADHD, attention‐deficit/hyperactivity disorder; ANX, anxiety; CUEs, commonly upsetting events; DEP, depression; EB, emotional burden; ERD, emotion regulation deficits; LD, linear discriminant function; LDA, linear discrimination analysis. ***p < .001, ****p < .0001

A significant group effect on EB Index (*F*[3,731] = 69.2, *p* < .001) was found, where the ADHD, autism and ADHD+autism groups, which did not differ from each other, had higher EB than the neurotypical peers (all *p*s < .001) (Figure 1A). The higher EB index in the ADHD and/or autism group relative to the neurotypical group remained after adjusting for differences in sex and race (white vs. nonwhite) across groups (*ps* < .001). Adjusting for sex also additionally revealed higher EB in the ADHD+autism than the ADHD group (*p* = .044).

### Association between EB index, ERD and mental health measures

Simple linear regressions revealed that EB (*b*_DEP_ = 0.60, 95% CI [0.54, 0.66]; *b*_ANX_ = 0.65, 95% CI [0.59, 0.70]), ERD (*b*_DEP_ = 0.69, 95% CI [0.63, 0.74]; *b*_ANX_ = 0.70, 95% CI [0.64, 0.75]), ADHD traits (*b*_DEP_ = 0.37, 95% CI [0.30, 0.44]; *b*_ANX_ = 0.40, 95% CI [0.34, 0.47]), autistic traits (*b*_DEP_ = 0.26, 95% CI [0.19, 0.33]; *b*_ANX_ = 0.28, 95% CI [0.21, 0.35]), alexithymia (*b*_DEP_ = 0.62, 95% CI [0.56, 0.69]; *b*_ANX_ = 0.64, 95% CI [0.58, 0.69]), separately predicted depression or anxiety (Standardised *b* reported; all *p*s < .001) (Table S9a). When ADHD and autism traits, EB, ERD and alexithymia were entered simultaneously in multivariate models for predicting either depression or anxiety, only EB (*b*_DEP_ = 0.29, 95% CI [0.22, 0.36]; *b*_ANX_ = 0.34, 95% CI [0.27, 0.41]), ERD (*b*_DEP_ = 0.34, 95% CI [0.31, 0.49]; *b*_ANX_ = 0.37, 95% CI [0.28, 0.45]) and alexithymia (*b*_DEP_ = 0.17, 95% CI [0.09, 0.25]; *b*_ANX_ = 0.18, 95% CI [0.10, 0.25]), but not ADHD or autism traits (*p*s > .29), remained predictive of these mental health difficulties (Table S9b). These patterns of findings were retained when we adjusted for sex and race differences across groups (Table S9c,d).

### ADHD‐ and autism‐specific CUEs

Our initial LDA findings involving all four groups (ADHD, autism, ADHD+autism and neurotypical) suggested that the ADHD+autism group was a heterogeneous group affected by CUEs found burdensome by the ADHD and autism alone groups (Appendix S2.8).

To increase the specificity of the finding, we therefore repeated the LDA including only the ADHD, autism and neurotypical group. We found that the EB indicators predicted group memberships with an overall accuracy of 0.76 95% CI [0.72, 0.79] and a ‘fair’ grouping reliability (*κ* = 0.35 *n*‐fold cross‐validation). The balanced accuracy of the grouping was 0.65 for ADHD, 0.66 for autism and 0.72 for the neurotypical group (confusion matrix Figure S6b).

The LDA model produced two linear discriminant (LD) functions explaining 81.7% and 18.3% of variance in the data (Figure 1C[i], Table 4). The comparison of the LD scores suggested that the first LD (LD1) differentiated both ADHD and autism from the neurotypical group (*F*[2,653] = 125.9; *p* < .001; ADHD, autism < neurotypical; Δ*M*_neurotypical‐ADHD_ = 1.39 [95% CI: 1.13, 1.65], Δ*M*_neurotypical‐autism_ = 1.28 [95% CI: 1.03, 1.54], both *p*s < .0001; Δ*M*_autism‐ADHD_ = 0.11 [95% CI: 0.22, 043], *ns*) (Figure 1C[ii]), with a few CUEs, that is *‘last‐minute change of plan’, ‘not allowed self‐regulation strategies’* and *‘being rushed to move on from task to task’* particularly burdensome for the ADHD or autism group (coefficients = −.39 to −.50) (Figure 1D, Table 4).

**Table 4:** LDA coefficients of EB indicators and their grouping influence

MESI CUEs	LD1 coefficients	LD2 coefficients	LD1 coefficient grouping influence	LD2 coefficient grouping influence
1. Peers talking behind my back	.261	−.324	Neurotypical (+)	Autism (++)
2. Unexpected wait in a queue	−.131	−.335	–	Autism (++)
3. Teachers tell me off	−.017	−.079	–	–
4. Schoolmates ignore me	.054	−.115	–	–
5. Teachers don't listen	−.103	.309	–	ADHD (++)
6. Teachers don't understand	−.275	−.192	Neurodivergent (+)	–
7. Last minute change of plan	−.393	−.054	Neurodivergent (++)	–
8. Not being able to do tasks	.169	−.038	–	–
9. Being in a chaotic classroom	−.229	−.013	Neurodivergent (+)	–
10. Boring lessons or tasks	.030	.314	–	ADHD (++)
11. Being stopped doing enjoyable things	.079	.337	–	ADHD (++)
12. Experiencing sensory discomfort	.257	−.502	Neurotypical (+)	Autism (++)
13. Losing and forgetting things	.063	.328	–	ADHD (++)
14. In trouble for losing or forgetting	−.056	−.097	–	–
15. Being rushed to complete work	−.039	−.314	–	Autism (++)
16. Not understanding others	−.269	−.302	Neurodivergent (+)	Autism (++)
17. Staff treating me unfairly	NA	NA	NA	NA
18. Peers teasing and bullying	−.019	−.254	–	Autism (+)
19. Being told to try harder	.150	.422	–	ADHD (++)
20. Being accused of something I didn't do	−.021	.411	–	ADHD (++)
21. Not doing something quite right	−.089	.207	–	ADHD (+)
22. Not allowed to do self‐regulation strategies	−.496	.076	Neurodivergent (++)	–
23. Being pressured to do well	.150	−.278	–	Autism (+)
24. Having too many options	.117	.265	–	ADHD (+)
25. Being rushed to move on	−.429	.063	Neurodivergent (++)	–

Linear discriminant (LD) functions, LD1 and LD2, describe 82% and 18% variance of the emotional burden (EB) data in the ADHD, autistic and neurotypical groups. LD1 distinguished both the ADHD and autistic groups collectively from the neurotypical group, while LD2 distinguished the ADHD from the autistic group. The absolute value of a coefficient >.3 denotes a CUE's strong influence (++) on the predicted diagnostic grouping. CUEs with coefficients >.2 are also sign‐posted (+) in the table. CUEs indexed neurodivergent exerted influences in the predicted grouping to the neurodivergent groups with ADHD or autism, while CUEs indexed neurotypical exerted influences in the predicted grouping to the neurotypical group. CUEs indexed autism or ADHD influenced the predicted grouping to the autism or ADHD group, respectively.

The less prominent LD2 differentiated all three groups, but primarily separated the ADHD from the autistic group (*F*[2, 653] = 28.2, *p* < .001; ADHD > neurotypical > autism; Δ*M*_ADHD‐neurotypical_ = 0.51 [95%CI: 0.25, 0.76], *p* < .001; Δ*M*_neurotypical‐autism_ = 0.55 [95%CI: 0.29, 0.80], Δ*M*_ADHD‐autism_ = 1.05 [95%CI: 0.72, 1.38]; both *p*s < .0001) (Figure 1C[iii]), driven by the CUEs *‘peers talking behind my back’, ‘unexpected wait in a queue’*, *‘sensory discomfort’*, *‘being rushed to complete work’ and ‘not understanding others’* were particularly burdensome for the autism group (coefficients = −.30 to −.50) and the CUEs *‘teachers don't listen’*, *‘boring lessons or tasks’*, *‘stopped from doing something enjoyable’*, *‘losing and forgetting things’*, *‘being told to try harder’* and *‘being accused of something I didn't do’* which were particularly burdensome for the ADHD group (coefficients = .31 to .42) (Figure 1D, Table 4).

These CUEs remained specifically burdensome for the respective groups after adjusting for either sex or race, although in some cases the magnitude of their coefficient was reduced from ≥.3 to between .2 and .3. An exception was the CUE *‘peers talking behind my back’* that was no longer especially burdensome for either the ADHD or the autism group after adjusting for sex differences across the groups (Table S10).

## Discussion

RE‐STAR is currently undertaking a longitudinal study to test the hypothesis that elevated EB, due to more frequent and more emotionally intensely experienced exposure to CUEs, plays a significant role in driving mental health risks experienced by adolescents with ADHD and/or autism, even after accounting for ERD. To prepare for this, academic researchers and young people with ADHD and/or autism co‐produced a novel and robustly tested self‐report measure, the MESI, which measured the frequency and intensity with which school‐experienced CUEs, reported by adolescents with ADHD and/or autism (Pavlopoulou et al., 2025) to be upsetting, were experienced. Our current analyses support the reliability and validity of the MESI EB Index, computed by summing the product of frequency and intensity of 24 CUE items within the MESI, and the value of measuring this index in the context of mental health in adolescents with ADHD and/or autism. There were several findings of note.

First, our initial analysis supports the importance of taking into account both a CUE's frequency and the intensity of the emotional response they induce in conceptualising emotional burden. Both variables were correlated strongly but not perfectly, indicating their sufficient difference, while overlapping to a degree, presumably due to their respective link to each unique CUE. Furthermore, adolescents with autism and/or ADHD reported greater frequency and intensity of CUEs than their neurotypical peers. This extends previous reports of increased frequency of exposure to daily stressors in the ADHD and/or autistic populations (Hartman, Rommelse, van der Klugt, Wanders, & Timmerman, 2019; Hoover & Kaufman, 2018; Khor, Melvin, Reid, & Gray, 2014; Öster, Ramklint, Meyer, & Isaksson, 2020) by providing another dimension of how intensely each CUE was experienced. Most importantly, CUE frequency and intensity were both uniquely associated with depression or anxiety. This suggests that the prediction/identification of mental health problems required both expressions of CUE frequency and intensity in the conceptualisation of emotional burden and our construction of the EB index.

Second, using the derived EB index we showed that adolescents with ADHD and/or autism reported substantially higher emotional burden at school than their neurotypical peers. Interestingly, a similar pattern of findings was observed across groups concerning mental health problems like depression, anxiety and measures of emotional dysregulation, underlining the specific emotional vulnerability of the ADHD and/or autistic groups relative to their neurotypical peers.

Third, despite total EB being similar across neurodivergent groups, closer examination at the individual CUE level identified distinctive sets of CUEs with discriminating burden among groups, specifically the ADHD and autistic groups. These sets of CUE include *‘teachers don't listen’*, *‘boring lessons or tasks’*, *‘being stopped doing enjoyable things’ and ‘being told to try harder’*, among others, which contributed mostly to the burden experienced by individuals in the ADHD group, perhaps reflecting the power of a conflictual relationship with authoritative figures to provoke an emotional reaction in those individuals; while the items ‘*peers talking behind my back’, ‘unexpected wait in a queue’*, *‘sensory discomfort’*, *‘being rushed to complete work’ and ‘not understanding others’* that represent ‘othering’ and physical environment were particularly burdensome for individuals in the autism group. It is interesting in this context that individuals with both ADHD+autism appeared to experience only the same level of burden as those with ADHD or autism alone – rather than the conditions acting additively. This was a surprising finding, which needs to be replicated in future studies. One possibility is that there exists a ‘ceiling’ in emotional burden experienced by neurodivergent individuals above which adding additional symptoms of ADHD to autism or autism to ADHD cannot exceed. This could be related specifically to limitations on the total number of school‐related upsetting events an individual can be exposed to in their daily lives. However, such an explanation is complicated by the fact that autistic and ADHD students rated different CUEs as upsetting. An alternative possibility is that the cooccurrence of ADHD and autism leads to the former cancelling out the latter effect and vice‐versa leading to each contributing to a smaller proportion of the elevation of emotional burden than each condition alone – such an explanation could be linked to differences in the intensity of emotional reactions – whereby autistic traits moderate the ADHD responses to certain CUEs and or vice‐versa. Future analyses, outside the scope of the current paper, are required to investigate these different hypotheses.

Fourth, although correlated with each other, EB and ERD independently contributed to cross‐sectional predictions of both anxiety and depression. Interestingly, while ADHD and autistic traits were predictive of anxiety and depression in simple regression analyses, their association with these mental health variables ceased when EB and ERD were included as predictors in the model. This suggests that, notwithstanding the potential importance of ERD, the novel concept of EB may add explanatory power to current models of the role of emotional dysregulation in pathways from ADHD and/or autism to mental health problems (e.g. Antony et al., 2022; Eyre et al., 2017; Sáez‐Suanes et al., 2020).

Longitudinal studies will be essential to unpick the mediating role of EB and ERD in the depression and anxiety risk in ADHD and/or autistic individuals. The reciprocal pathways between mental problems and emotional regulation, and more specifically, between ERD and EB, need to be explored. One hypothesis worthy of study is that EB creates ERD over time – in a way that is consistent with the notion that adverse exposures can disrupt emotion regulation in fundamental ways (e.g. Miu et al., 2022) that may extend to cognitive difficulties in emotional processing and control. Furthermore, depression and anxiety can reduce social and psychological resources and generate stress that could have an exacerbating effect – increasing CUEs and their impacts and reducing self‐regulation capacity. RE‐STAR is currently exploring these processes in a 12‐month longitudinal study called My Emotions and Me Over Time (Sonuga‐Barke et al., under review).

From a clinical perspective, the concept of induced EB has the potential to expand, or even to shift, the focus of emotion‐related interventions from attempts to reduce ERD within an individual through emotion regulation training alone (e.g. Carroll, Hirvikoski, Lindholm, & Thorell, 2023; Zaharia et al., 2021) to ones that incorporate EB reduction – by decreasing CUEs and/or improving ways such provocations might be managed to ameliorate the intensity of emotional reactions they induce.

The strengths of the investigation include the large sample size, exceeding our initial sample size estimation, which ensured adequate power for the psychometric analysis. The overall participants demographic included diverse ethnic backgrounds, and a sizeable proportion of neurodivergent (defined either by a diagnosis or as meeting cut‐offs on clinically validated instruments; 38.5%) and female participants (36%), among whom the robustness of our EB Index has been demonstrated. That said, white ethnicity was the substantial majority in the ADHD and/or autism groups, which is nonrepresentative of the ethnic landscape of the population (see e.g. Roman‐Urrestarazu et al., 2021), and in the absence of extensive clinical assessments, neurodivergences other than autism and/or ADHD may be part of the make‐up of the ‘neurotypical’ group. Nevertheless, differences in sex and ethnicity across groups exerted minimal influence on our findings. Further, our focus on mainstream school attendees would preclude the investigation of emotional burden among school absentees, a proportion of whom may have ADHD and/or autism (Nordin, Palmgren, Lindbladh, Bölte, & Jonsson, 2024), although these individuals arguably require more complex support needs and dedicated investigation. Finally, concurrent EB measurements in settings other than school, although beyond the scope of MESI, could be useful in future studies for monitoring their potential influences on the reporting of school‐based EB.

To conclude, the current paper demonstrates the reliability and validity of the EB index as measured by the MESI, co‐produced by academic and youth researchers with ADHD and/or autism. If the current analyses are confirmed in longitudinal studies, EB could add significant power to explain the emergence of mental health problems in adolescents with ADHD and/or autism, over and above more traditional measures of emotion dysregulation. By doing this, it has the potential to provide a new way of thinking about preventative interventions that might reduce mental health risks for neurodivergent individuals.

## Ethical information

The study was approved by NHS Health and Social Care Research Ethics Committee (HSC REC A; REC reference 23/NI/0047) and was conducted in accordance with the Declaration of Helsinki. Informed consent/assent was obtained as appropriate from all participants before their participation.

**Key points**

- Emotion dysregulation has been implicated in depression risk in neurodivergent adolescents.
- In RE‐STAR we are comparing Emotion Regulation Deficits (ERD) and Emotional Burden (EB) as potential mechanisms underpinning this risk.
- The ‘My Emotions in School Inventory (MESI)’ was co‐produced with young people with ADHD and/or autism to measure levels of emotional burden (EB – combining frequency of exposure to common upsetting events and intensity of emotional responses to them) in adolescents.
- EB levels were higher in the autism and/or ADHD groups, compared to the neurotypical group.
- EB and ERD were associated with mental health problems, independent of each other.
- Specific events provoking EB were different for ADHD and autistic adolescents.
- The concept of EB, as measured by the MESI, could have implications for interventions that aim to promote the wellbeing of autistic young people and those with ADHD.

## Supporting information


**Appendix S1.** Supporting methods.
**Appendix S2.** Supporting results.

## Data Availability

The data that support the findings of this study are available from the corresponding author upon reasonable request.

## Appendix A

The RE‐STAR team is: Edmund Sonuga‐Barke, Susie Chandler, Andrea Danese, Eloise Funnell, Johnny Downs, Kirsty Griffiths, Myrofora Kakoulidou, Lauren Low, Steve Lukito, Umaya Prasad, Angus Roberts, Emily Simonoff, Daniel Stahl, Anna Wyatt (King's College London); Georgia Pavlopoulou (Anna Freud Centre and University College London [UCL]) and Jane Hurry (UCL); Sylvan Baker (Royal Central School of Speech and Drama); Graham Moore (Cardiff University); Dennis Ougrin (Queen Mary University of London); Amanda Roestorf (Autistica), Rebecca Kirkbride (Place2Be); Claire Lewis (ADHD Foundation) and Youth Researcher Panel members Jordan Altimimi, Beta Balwani, Saskia Barnes, Tiegan Boyens, Zoë Glen, Cj Harris, Charlotte Hillman, Luke Harvey‐Nguyen, Issy Jackson, Amber Johnson, Elisa Ly, Maciej Matejko, Dorian Poulton, Anya Rose, Darren Webb, Archie Wilson.
