# Supplementary material for: Emotional burden in school as a source of mental health problems associated with ADHD and/or autism: Development and validation of a new co‐produced self‐report measure
Source: J Child Psychol Psychiatry. 2025 Jul 24;66(10):1577–92. doi: 10.1111/jcpp.70003 (PMC12447693; doi:10.1111/jcpp.70003)
Supplement: Supplementary file 1 — Appendix S1. Supporting methods. Appendix S2. Supporting results. [file JCPP-66-1577-s001.docx]

**Supporting information to Lukito, Chandler et al. (2024) Emotional burden in school as a source of mental health problems in adolescents with ADHD and/or autism: Development and validation of a new co-produced self-report measure.**

**Table of Content**

| **Appendix S1. Supporting Methods** | 2 |
| --- | --- |
| S1.1. RE-STAR Co-production Stages | 2 |
| S1.2. Piloting Stage of the My Emotions in School Inventory (MESI) Prototype | 3 |
| S1.3. Final MESI | 3 |
| S1.4. Sample Size Estimation | 4 |
| S1.5. Statistical Analysis Plan | 4 |
| S1.5.1. Preliminary Item Analysis | 4 |
| S1.5.2. Scale Development | 4 |
| S1.5.3. Measurement Equivalence Analysis | 6 |
| S1.5.4. Linear Discriminant Analysis (LDA) | 6 |
| S1.5.5**.** Sensitivity Analyses Adjusting for Sex and Race | 6 |
| Appendix S2. Supporting Results | 6 |
| S2.1. Piloting Stage of the MESI Prototype | 7 |
| S2.2. Preliminary Item Analysis | 8 |
| S2.3. Descriptives of MESI CUE Frequency and Intensity Variables | 8 |
| S2.4. Scale Development | 12 |
| S2.5. Measurement Equivalence | 15 |
| S2.6. Differences of EB Index Across the Diagnostic Groups: Sensitivity Analyses | 16 |
| S2.7. Associations between EB Index, ERD, and Mental Health Measures: Sensitivity Analyses | 16 |
| S2.8. Additional LDA Results and Sensitivity Analyses | 17 |
| S2.8.1. Additional Four-Group LDA Results | 17 |
| S2.8.2. Sensitivity Analyses of the LDA Adjusted for Sex and Race | 19 |

# Supporting Methods

## RE-STAR Co-production Stages

In addition to the information presented in the main paper, we summarise the step-by-step processes of co-production of the MESI on Fig S1.

**Figure S1. Stages of development for MESI items**

| **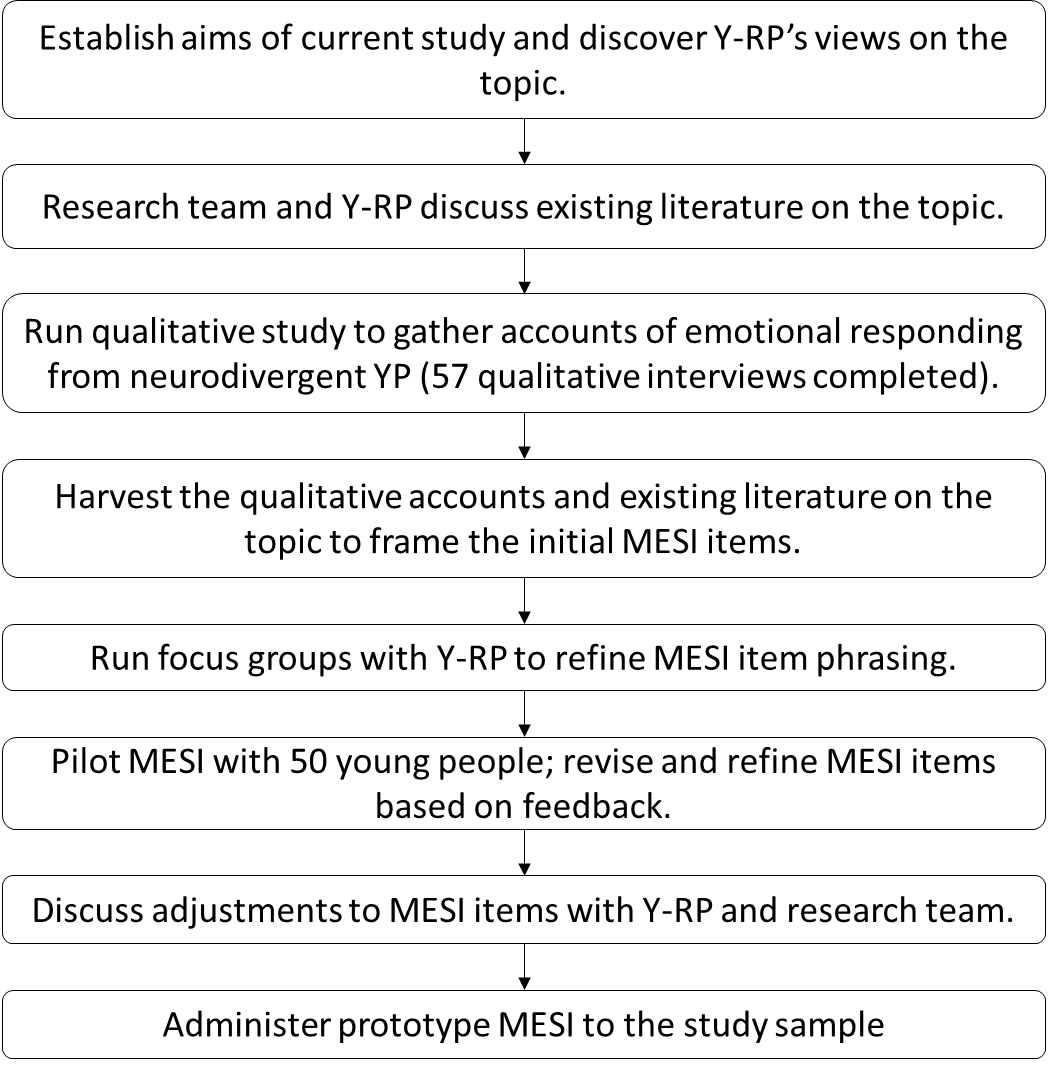** |
| --- |

Abbreviations: MESI = My Emotions in School Inventory; YP = Young people; Y-RP = Youth Researcher Panel

## Piloting Stage of the My Emotions at School Inventory (MESI) Prototype

The prototype of MESI consisted of 17 CUEs rated according to: (a) the *frequency* of the upsetting event, (b) the *likelihood* it would upset them, and (c) *how much* or the *intensity* with which it would upset them. Each item was rated on a 9-point Likert scale. Each scale was marked by 5 response anchors: (a) 1=*never*, 3=*rarely*, 5=*sometimes*, 7=*often*, and 9=*frequently* for the CUE frequency items, (b) 1=*very unlikely*, 3=*unlikely*, 5=*possibly*, 7=*likely*, and 9=*very likely* for the CUE likelihood items, and (c) 1=*not at all*, 3=*a little*, 5=*somewhat*, 7=*a lot*, and 9=*extremely* for the CUE intensity items; with the possibility for participants to characterise their experiences between two response anchors. A text box was provided at the end of the questionnaire to gather free-text feedback and comments from the participants. This feedback and item adjustments were discussed with the Y-RP members.

This prototype measure was piloted with 50 young people aged 11-16 who attended mainstream secondary school and had a diagnosis of ADHD (n=19), autism (n=21), or ADHD+autism (n=10). Forty-two (84%) participants took part in the qualitative interview stage (Table S1). Feedback from the participants was examined. We then inspected the distributions of item responses for each CUE on histograms and examined the pairwise correlations between items.

**Table S1. Pilot-stage Participant Characteristics**

| Variables | Overall | | ADHD | | autism | | ADHD+autism | |
| --- | --- | --- | --- | --- | --- | --- | --- | --- |
|  | (N=50) | | (N=19) | | (N=21) | | (N=10) | |
| Age, years (M, SD) | 13.4 | 1.42 | 13.4 | 1.64 | 21 | 1.17 | 13.9 | 1.45 |
| Sex, female (n [%]) | 22 | (44.0) | 6 | (31.6) | 13 | (61.9) | 3 | (30.0) |
| Ethnicity (n [%]) |  |  |  |  |  |  |  |  |
| White | 40 | (80.0) | 16 | (84.2) | 17 | (81.0) | 7 | (70.0) |
| Black British/ African/Caribbean | 3 | (6.0) | 0 | (0.0) | 1 | (4.8) | 2 | (20.0) |
| Asian /Asian British | 1 | (2.0) | 0 | (0.0) | 0 | (0.0) | 1 | (10.0) |
| Other ethnic group | 6 | (12.0) | 3 | (15.8) | 3 | (14.3) | 0 | (0.0) |
| Receive free school meals (n [%]) | 9 | (18.0) | 5 | (26.3) | 2 | (9.5) | 2 | (20.0) |

## Final MESI

The final MESI contains 25 commonly upsetting events (CUEs) (Table S1). Each CUE is rated on five-point Likert scale on (a) the frequency of upsetting events (i.e., ‘frequency’ measure [0=*never*, 1=*rarely*, 2=*sometimes*, 3=*often*, 4=*frequently*]), and (b) how much upset each event would cause (i.e., ‘intensity’ measure [0=*not at all*, 1=*a little*, 2=*somewhat*, 3=*a lot*, to 4=*extremely*]). Therefore, each CUE produces a pair of frequency and intensity variables that are subsequently used to develop the EB index. The frequency and intensity variables are shown for the first CUE only in Table S1.

**Table S2. My Emotions at School Inventory**

| Here is a list of things that could happen at school that might be upsetting for children. Can you tell us (a) how often this has happened to you, and (b) how upset you would be if it happened today? | | | | | |
| --- | --- | --- | --- | --- | --- |
| 1. | Finding out your peers have been talking about you behind your back. | | | | |
|  | Has this happened to you? | | | | |
|  | Never | Rarely | Sometimes | Often | Frequently |
|  |  |  |  |  |  |
|  | If this happened today, how much would it upset you? | | | | |
|  | Not at all | A little | Somewhat | A lot | Extremely |
|  |  |  |  |  |  |
| 2. | Unexpectedly having to wait for ages in a queue. | | | | |
| 3. | Being told off by your teacher in front of your classmates. | | | | |
| 4. | Schoolmates don’t listen to what you say or ignore you. | | | | |
| 5. | School staff don’t listen to you or challenge what you say. | | | | |
| 6. | School staff don’t understand your feelings and reactions. | | | | |
| 7. | The person in charge makes a last-minute change of plan. | | | | |
| 8. | Not being able to do a task at school. | | | | |
| 9. | Being in a chaotic classroom and/or playground (e.g., too noisy, too many people around you, too many visuals). | | | | |
| 10. | When you’re asked to do something really boring. | | | | |
| 11. | Being made to stop doing something you really enjoy by school staff. | | | | |
| 12. | The sights, smells, or sounds in the classroom make you feel uncomfortable. | | | | |
| 13. | Losing or forgetting something important for your lessons (e.g., your school bag or physical education [PE] kit). | | | | |
| 14. | Getting into trouble for losing and/or forgetting your stuff (e.g., homework). | | | | |
| 15. | Being rushed to complete some work. | | | | |
| 16. | Not ‘getting’ or understanding what others are talking about. | | | | |
| 17. | School staff treating you unfairly (e.g., by giving an unnecessary detention). | | | | |
| 18. | Being teased and/or bullied by peers. | | | | |
| 19. | Others telling you to try harder when you’ve already tried your best. | | | | |
| 20. | Being accused of something you didn’t do. | | | | |
| 21. | Not being able to get something ‘quite right’ (e.g., a drawing, piece of schoolwork, computer game). | | | | |
| 22. | Not being allowed to do the things that help you manage your emotions (e.g., use fidget toys, get out of your seat, leave the classroom). | | | | |
| 23. | Feeling pressure to do well (e.g., in exams, getting homework done, getting a high school grade). | | | | |
| 24. | Having too many options to choose from. | | | | |
| 25. | Being rushed to move from one thing to another. | | | | |

Note. The final MESI consisted of 25 school-based commonly upsetting events and were rated on the frequency and intensity on a 5-point Likert Scale (frequency and intensity ratings are only displayed for the first CUE).

## Sample Size Estimation

For the scale development purpose, a minimum sample size of n=600 was estimated for an EFA involving up to 60 MESI frequency and intensity variables. Sample size requirements to reliably identify a replicable factor structure depend on a variety of factors, among others, loading and number of factors, which cannot be known at the planning stage (de Winter, Dodou, & Wieringa, 2009). Up to two latent factors were probable for MESI (i.e., onto which the frequency and intensity items separately load). Simulations have shown that a 2-factor structure with 48 items and moderate loadings can be reliably reconstructed with a sample size of 112, but this increases with distortions of the ideal structure (de Winter et al., 2009). The minimum sample size of 600 is in agreement with the rules of thumb, i.e., 10 respondents per variable (Nunnally, 1978, p. 421), which we deemed most suitable estimation for our study, where no a priori measurement model (e.g., item framing or factor structure) was assumed.

## Statistical Analysis Plan

### Preliminary Item Analysis

As specified in the main text, the preliminary item analysis was conducted on the 50 CUE frequency and intensity variables of the MESI, to establish if these variables: (a) were *not* scored the same way by >80% of participants, which indicated that they had sufficient rating variation across individuals, (b) demonstrated no signs of separate distributions across subgroups of participants, e.g., bimodal across subgroups of neurotypical or neurodivergent, (c) were not pairwise intercorrelated above *r*>0.8 and had at least one correlation with *r*>0.3 with other items, i.e., each variable shares some characteristics with other items due to the common latent factor they measure but none of the variables were redundant because they measure the exact characteristic of another variable (see Streiner & Norman, 2008), and, finally, (d) had less than 5% missing responses, i.e., the items were scoreable by most participants without difficulty. The CUE items were considered for removal if their frequency and/or intensity did not meet those criteria at this stage. For completion, these preliminary item analyses were applied to indicators of the EB latent factor (i.e., frequency × intensity).

### Scale Development

As described in the main paper, the latent factor EB was first conceptualised and indicated by the combination of CUE intensity and frequency (Fig S2). Each EB indicator was the multiplication of each CUE frequency and its intensity, which was a favoured approach for combining data since it preserves rating order (Ajzen & Fishbein, 2008; Amon, Annand, & Holden, 2022; Tofallis, 2014), and ensures zero burden when no CUE occurs or when an individual feels no upset despite experiencing frequent CUEs.

The Scale Development stages in the main paper described the procedures to produce a robust and parsimonious measurement model of EB, which included an assessment of the factor structure of EB using exploratory factor analysis (EFA). Scree plots (Cattell, 1966) and parallel analyses (Horn, 1965; Pearson, Mundfrom, & Piccone, 2013) helped identify the optimal number factor.

After the final measurement model of EB was achieved (Fig S2), we created the *EB Index*, a sum of the multiplication of frequency and intensity (Equation 1) of the CUEs included in the measurement model of the EB latent factor, i.e., those CUE remaining after indicator removal procedures were completed (indicated below by the subscript *i*).

| $EB\mathrm{Index}= \sum_{i} F_{i} \times I_{i}$ | Equation (1) |
| --- | --- |

**Figure S2. EB Latent Factor Measurement Model**

| 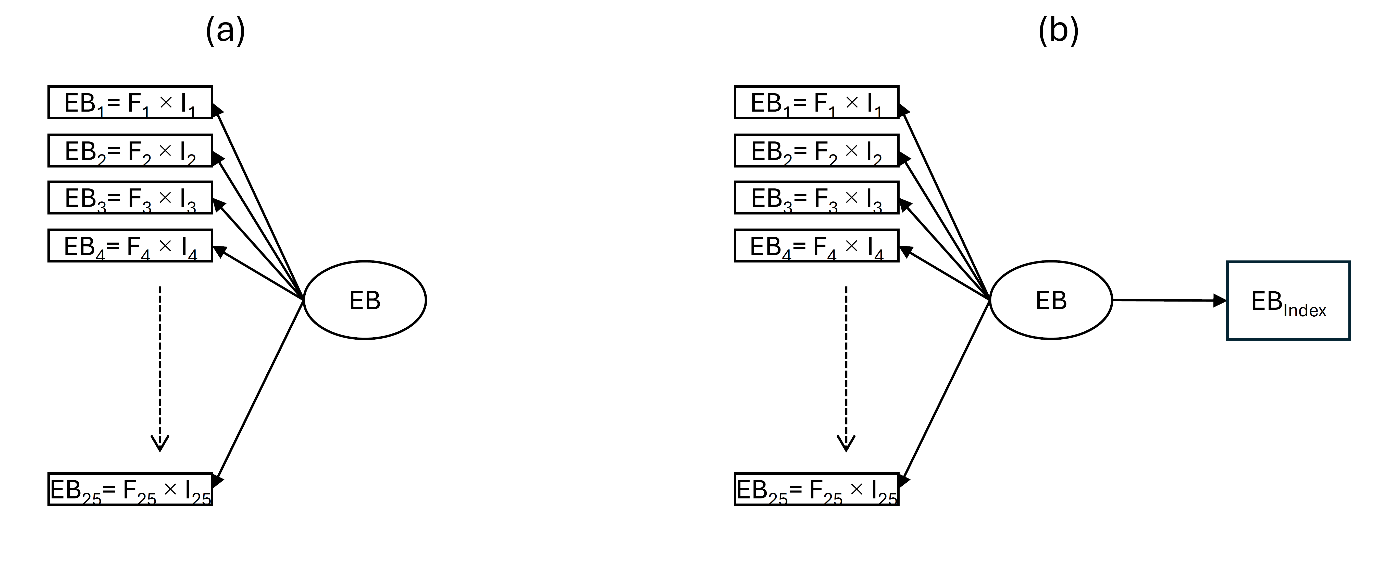 |
| --- |
| Note. The latent factor emotional burden (EB) is indicated by the EB indicator posed by each commonly upsetting events (CUE), defined as the CUE frequency multiplied by its intensity. |

The internal consistency of the EB indicator was investigated using Cronbach’s α and item-total correlation, removing one indicator from at a time, when the indicator’s influence on these internal consistency metrics was investigated.

We investigated how good the EB index was as a proxy score for representing the EB latent factor: First, we examined the relative agreement of the EB index as a proxy of the EB latent factor, by correlating the scaled EB index and the extracted value of the EB latent factor using ICC across participants. Second, to assess the unbiased nature of the EB index as a proxy for the EB factor, we repeated the ICC analyses across subgroups of diagnosis and sex (i.e., ICC >0.9).

### Measurement Equivalence Analysis

As an additional analysis of the robustness of the best-fitting measurement model of EB, we examined the measurement equivalence or invariance of the EB latent factor using a series of multi-group confirmatory factor analyses (MGCFA) to see the consistency of the relationship between the CUE-specific EB indicators and the EB latent factor across subgroups of participants, defined by their diagnosis, sex, and race. To allow powered analysis, we reconfigured ethnicity into race (white vs. non-white) for the latter analysis due to the relatively low number of participants in some ethnic subgroups in the original categories (i.e., White, Black British, Asian British, Mixed Race, and Other ethnic groups). Specifically, we examined: (1) the configural invariance, i.e., whether the same measurement models were applicable across groups, (2) the metric invariance, i.e., whether the factor loadings of the measurement model were the same across groups, and (3) the scalar invariance, i.e., whether the latent mean scores can be compared between groups without bias.

### Linear Discriminant Analysis (LDA)

As outlined in the main paper, we compared the EB carried across groups using Analyses of Variance (ANOVA). In addition to this analysis, we were interested in finding out if some subsets of CUEs were particularly burdensome for specific diagnostic groups (e.g., in ADHD vs autism). We used linear discriminant analyses (LDA) to investigate this. The LDA was conducted using the EB indicators as predictors of the diagnostic groups. We conducted the LDA, first, with all four diagnostic groups included (results presented here), and second, with the neurotypical, autistic, and ADHD groups only to improve specificity (main manuscript). The grouping reliability was indexed with Kappa statistics computed using n-fold cross-validation procedures (Hastie, Tibshirani, & Friedman, 2009). The interpretation of the LDA finding was aided by the comparison of scores of the discriminant functions, which were statistically tested using ANOVA. Coefficients of the discriminant functions associated with each EB indicator were also examined, where EB indicators with an absolute coefficient value >.30 were deemed to be influential to specific groups (Dhamnetiya, Goel, Jha, Shalini, & Bhattacharyya, 2022; Lambert & Durand, 1975). We confirmed our analyses using the robust LDA approach.

### Sensitivity Analyses Adjusting for Sex and Race

We conducted additional sensitivity analyses, adjusting for the varying sex, and race differences across the ADHD, autism, ADHD+autism, and neurotypical groups. Due to the low number of participants in some ethnic subgroups, ethnicity was again reconfigured into race (white vs. non-white) subgroups. The sensitivity analyses were applied to our investigation of group differences, analyses of associations among EB index, emotional regulation deficits (ERD), and mental health measures, and also the linear discriminant analyses (LDA).

The sensitivity analyses of group differences of EB index were investigated using 2(sex)*4(groups according to ADHD and/or autism traits) ANOVA, and using 2(race - white vs. non-white)*4(groups according to ADHD and/or autism traits) ANOVAs. Pairwise differences between groups were analysed *post-hoc* with TukeyHSD corrections applied to account for multiple comparisons. The sensitivity analyses of associations among EB index, ERD and mental health measures were conducted by adding the variables sex and race in the multiple regression model. Lastly, we adjusted sex and race in the LDA by regressing the EB indicator from each CUE on sex or race, and entering residuals of each EB indicator as predictors of the LDA model and proceeded to complete the LDA stages as outlined in the previous section.

# Supporting Results

## Piloting Stage of the MESI Prototype

Feedback for the MESI prototype revolved around: (1) the need for clearer wordings for the items (n=7) (e.g., “*I didn't understand a few words and questions*”, “*I had to ask my mum to explain a couple of things*”, “*Better wording with questions/ more variety of words*”) (2) the possibility of adding own writings (e.g., “*Option for free text answers*”, “*You could add like your own answer instead of needing to use them answers*”, and (3) the length of and repetitiveness of questions (n=4) (e.g., “*I thought bit was OK, maybe a little long”*, “*Could be a little bit shorter*”, “*Feel less repetitive*”, *“Less options”*), that our participants marked as potential areas for improvement. Wording issues were worked on with the Y-RP members in the subsequent stage.

Visual examinations of the item response histograms indicated that the participants’ ratings were frequently concentrated around the anchors of the Likert scale. The participants are less likely to rate their experience between two response anchors (Fig S3). Thus, we simplified our response format from 9-point to 5-point Likert scale, while retaining the labels of the original response anchors for the 5-point scale.

**Figure S3. Item Response Histograms**

| **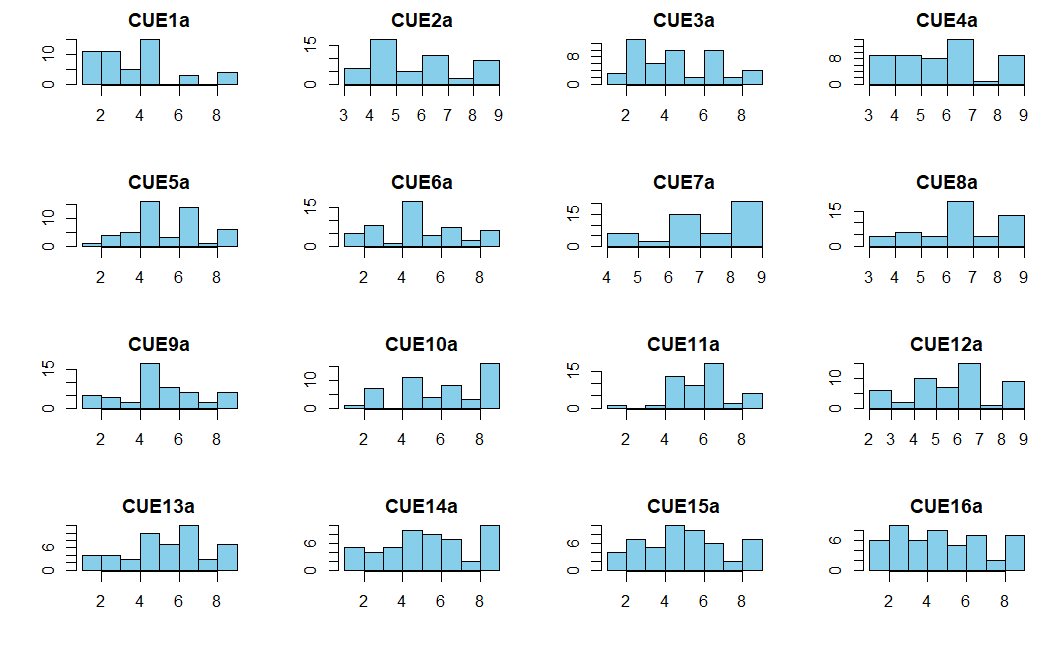** |
| --- |
| Note. Example response distribution for the CUE measure (plotted for the frequency measure on histograms for some MESI prototype questionnaire items). Similarly jagged histogram features were observed for the likelihood and intensity measures |

Within the same CUEs, pairwise *Pearson’s* correlations between the frequency (a) and likelihood measures (b) ranged between -0.39 and 0.45 (mean *r*=0.14; SD=0.20). The pairwise correlations between the frequency (a) and intensity items (c) ranged between -0.38 and 0.42 (mean *r*=0.13; SD=0.22). In contrast, the pairwise correlations between the likelihood (b) and the intensity (c) items ranged between 0.64 and 0.93 (mean *r*=0.83; SD=0.08), indicating that autistic and ADHD participants rated the likelihood and intensity of upset highly similarly within the same CUE (Fig S4), i.e., one of these items was redundant. Feedback from the Y-RP members regarding the abstract nature of the likelihood items supported the eventual decision to drop them. We kept the intensity items, that can be answered concretely by our participants.

Finally, we reviewed our previous consultations with the Y-RP members and the reflexive themes that have emerged from the qualitative study of the interviews previously conducted with our participants. Driven by this reflection, we decided to focus our investigation of the EB of CUEs in the school context by conducting some revisions or wordings and adding 8 school-specific CUEs in the MESI.

**Figure S4. The Histograms of Correlation Indices among the CUE Frequency, and the Likelihood and Intensity of Upset**

Note. Pairwise correlations between the frequency (a) and likelihood item (b) (coloured blue), between the frequency (a) and intensity (c) items (coloured green), and between the likelihood (b) and the intensity items (c) (coloured yellow) within the same CUE. Participants appeared to rate the likelihood and intensity of upset of each CUE highly similarly.

## Preliminary Item Analysis

A summary of the preliminary item analysis is presented in Table S3. None of the 50 frequency and intensity variable ratings were rated similarly by more than 80% of participants (range: 23.6-39.3%). Data were not distributed bimodally across variables. No two variables correlated with Pearson’s *r* > 0.8 (range: -0.038-0.69) and all variables had at least one pairwise correlation *r*>0.3 with another. For completion, we also conducted the analyses with the indicators (i.e., frequency x intensity) of EB latent factor before the scale development and similarly all indicator variable ratings satisfied the preliminary item analysis criteria (Table S3). Therefore, all CUEs were included in the subsequent exploratory factor analysis.

**Table S3. Preliminary Item Analysis Finding Summary**

|  | All frequency and intensity variables | EB indicator variables |
| --- | --- | --- |
| Items similarly scored by >80% of participants | None  (Range: 23.6-39.3%) | None  (Range: 17.2-41.8%) |
| Bimodally distributed variables | No | No |
| Correlation of r>0.8 between variables | None  (Range r: -0.038-0.69) | None  (Range r: 0.21-0.74) |
| All variables have at least one correlation r > 0.3 with another variable | Yes  (Range: 5-36) | Yes  (Range: 1-8) |
| Percent missing responses per variable (%) | Range: 0-0.5% | Range: 0-0.5% |
| Note. Finding summary of preliminary item analysis, applied to all frequency and intensity variables and to the EB indicator variables (i.e., frequency x intensity) | | |

## Descriptives of MESI CUE Frequency and Intensity Variables

The mean, 95%CI, and mean rank of the frequency and intensity variables in the overall sample and the ADHD, autism, ADHD+autism, and neurotypical groups are presented in Table S4. The CUEs are presented in the Table in a shorthand form.

**Table S4. Mean and Mean Rank of the CUE by Frequency, Intensity and Frequency x Intensity**

|  | Overall (N=735) | | | ADHD (n=100) | | | autism (n=104) | | | ADHD+autism (n=79) | | | neurotypical(n=452) | | |
| --- | --- | --- | --- | --- | --- | --- | --- | --- | --- | --- | --- | --- | --- | --- | --- |
|  | M | 95% CI | Rank | M | 95% CI | Rank | M | 95% CI | Rank | M | 95% CI | Rank | M | 95% CI | Rank |
| 1. CUE FREQUENCY | | | | | | | | | | | | | | | |
| Peers talking behind my back | 1.38 | [1.29, 1.46] | 23 | 1.52 | [1.29, 1.75] | 24 | 1.72 | [1.48, 1.97] | 19 | 1.80 | [1.48, 2.11] | 25 | 1.19 | [1.10, 1.29] | 19 |
| Unexpected wait in a queue | 2.14 | [2.06, 2.22] | 4 | 2.33 | [2.12, 2.54] | 9 | 2.25 | [2.05, 2.45] | 6 | 2.37 | [2.11, 2.63] | 9 | 2.04 | [1.94, 2.13] | 4 |
| Teachers tell me off | 1.70 | [1.61, 1.78] | 14 | 2.55 | [2.30, 2.80] | 3 | 1.61 | [1.42, 1.79] | 24 | 2.16 | [1.89, 2.44] | 16 | 1.44 | [1.35, 1.54] | 15 |
| Schoolmates ignore me | 1.62 | [1.54, 1.70] | 16 | 1.76 | [1.55, 1.97] | 22 | 1.93 | [1.71, 2.15] | 14 | 2.06 | [1.78, 2.35] | 20 | 1.45 | [1.35, 1.54] | 14 |
| Teachers don't listen | 1.43 | [1.34, 1.52] | 22 | 2.12 | [1.87, 2.37] | 19 | 1.68 | [1.43, 1.93] | 22 | 2.18 | [1.89, 2.46] | 15 | 1.09 | [0.99, 1.19] | 23 |
| Teachers don't understand | 1.54 | [1.44, 1.64] | 19 | 2.33 | [2.06, 2.60] | 10 | 2.07 | [1.81, 2.32] | 12 | 2.24 | [1.95, 2.53] | 14 | 1.12 | [1.01, 1.23] | 22 |
| Last minute change of plan | 1.71 | [1.64, 1.79] | 13 | 2.18 | [2.00, 2.36] | 18 | 2.09 | [1.87, 2.30] | 10 | 2.28 | [2.04, 2.52] | 13 | 1.43 | [1.34, 1.51] | 16 |
| Not being able to do tasks | 1.81 | [1.73, 1.89] | 10 | 2.24 | [2.01, 2.47] | 14 | 2.08 | [1.86, 2.29] | 11 | 2.42 | [2.16, 2.68] | 6 | 1.55 | [1.45, 1.64] | 9 |
| Being in a chaotic classroom | 2.53 | [2.44, 2.62] | 1 | 2.94 | [2.73, 3.15] | 1 | 2.85 | [2.62, 3.07] | 1 | 2.95 | [2.70, 3.20] | 1 | 2.29 | [2.17, 2.40] | 3 |
| Boring lessons or tasks | 2.50 | [2.42, 2.58] | 2 | 2.92 | [2.74, 3.10] | 2 | 2.60 | [2.38, 2.82] | 3 | 2.81 | [2.60, 3.02] | 2 | 2.33 | [2.24, 2.43] | 2 |
| Being stopped doing enjoyable things | 1.72 | [1.64, 1.80] | 12 | 2.25 | [2.04, 2.46] | 13 | 1.76 | [1.54, 1.98] | 18 | 2.11 | [1.85, 2.38] | 19 | 1.52 | [1.42, 1.63] | 10 |
| Experiencing sensory discomfort | 1.43 | [1.34, 1.52] | 21 | 1.66 | [1.42, 1.90] | 23 | 1.98 | [1.72, 2.24] | 13 | 2.06 | [1.77, 2.36] | 21 | 1.15 | [1.04, 1.25] | 20 |
| Losing and forgetting things | 1.70 | [1.61, 1.78] | 15 | 2.26 | [2.02, 2.50] | 12 | 1.85 | [1.63, 2.06] | 15 | 2.14 | [1.87, 2.41] | 18 | 1.46 | [1.37, 1.55] | 12 |
| In trouble for losing or forgetting | 1.54 | [1.46, 1.63] | 18 | 2.20 | [1.95, 2.45] | 16 | 1.60 | [1.37, 1.82] | 25 | 2.16 | [1.87, 2.46] | 17 | 1.28 | [1.18, 1.37] | 18 |
| Being rushed to complete work | 2.04 | [1.96, 2.13] | 6 | 2.47 | [2.26, 2.68] | 4 | 2.25 | [2.06, 2.44] | 7 | 2.42 | [2.17, 2.67] | 7 | 1.84 | [1.73, 1.94] | 6 |
| Not understanding others | 1.91 | [1.83, 1.99] | 7 | 2.20 | [1.99, 2.41] | 17 | 2.38 | [2.18, 2.59] | 4 | 2.33 | [2.09, 2.57] | 10 | 1.67 | [1.57, 1.76] | 7 |
| Staff treating me unfairly | 1.45 | [1.36, 1.54] | 20 | 2.23 | [1.95, 2.51] | 15 | 1.64 | [1.39, 1.90] | 23 | 2.01 | [1.69, 2.33] | 23 | 1.13 | [1.03, 1.24] | 21 |
| Peers teasing and bullying | 1.26 | [1.17, 1.35] | 25 | 1.41 | [1.17, 1.65] | 25 | 1.69 | [1.46, 1.93] | 20 | 1.86 | [1.53, 2.19] | 24 | 1.02 | [0.92, 1.12] | 24 |
| Being told to try harder | 1.57 | [1.48, 1.66] | 17 | 2.04 | [1.80, 2.28] | 21 | 1.69 | [1.47, 1.91] | 21 | 2.29 | [1.99, 2.59] | 12 | 1.31 | [1.21, 1.42] | 17 |
| Being accused of something I didn’t do | 1.73 | [1.64, 1.81] | 11 | 2.38 | [2.14, 2.62] | 8 | 1.81 | [1.57, 2.04] | 16 | 2.33 | [2.03, 2.63] | 11 | 1.46 | [1.36, 1.55] | 13 |
| Not doing something quite right | 2.05 | [1.97, 2.13] | 5 | 2.40 | [2.22, 2.58] | 6 | 2.28 | [2.07, 2.48] | 5 | 2.49 | [2.26, 2.73] | 4 | 1.85 | [1.75, 1.94] | 5 |
| Not allowed to do self-regulation strategies | 1.27 | [1.18, 1.37] | 24 | 2.10 | [1.83, 2.37] | 20 | 1.77 | [1.52, 2.02] | 17 | 2.05 | [1.75, 2.35] | 22 | 0.84 | [0.74, 0.94] | 25 |
| Being pressured to do well | 2.46 | [2.36, 2.55] | 3 | 2.39 | [2.14, 2.64] | 7 | 2.64 | [2.38, 2.91] | 2 | 2.58 | [2.29, 2.88] | 3 | 2.41 | [2.29, 2.52] | 1 |
| Having too many options | 1.87 | [1.78, 1.95] | 8 | 2.28 | [2.05, 2.51] | 11 | 2.16 | [1.93, 2.39] | 9 | 2.38 | [2.13, 2.63] | 8 | 1.62 | [1.51, 1.72] | 8 |
| Being rushed to move on | 1.81 | [1.73, 1.89] | 9 | 2.42 | [2.21, 2.63] | 5 | 2.21 | [2.03, 2.40] | 8 | 2.43 | [2.18, 2.68] | 5 | 1.48 | [1.39, 1.57] | 11 |
| 1. CUE INTENSITY | | | | | | | | | | | | | | | |
| Peers talking behind my back | 1.84 | [1.75, 1.92] | 12 | 1.82 | [1.58, 2.06] | 21 | 2.19 | [1.95, 2.43] | 19 | 2.18 | [1.88, 2.47] | 17 | 1.70 | [1.60, 1.81] | 9 |
| Unexpected wait in a queue | 1.46 | [1.38, 1.55] | 22 | 1.84 | [1.58, 2.10] | 20 | 2.03 | [1.78, 2.27] | 22 | 2.20 | [1.92, 2.48] | 16 | 1.12 | [1.03, 1.22] | 23 |
| Teachers tell me off | 1.69 | [1.59, 1.78] | 17 | 1.62 | [1.36, 1.88] | 24 | 2.41 | [2.16, 2.67] | 11 | 2.15 | [1.84, 2.46] | 19 | 1.45 | [1.34, 1.57] | 14 |
| Schoolmates ignore me | 1.71 | [1.63, 1.80] | 15 | 1.94 | [1.69, 2.19] | 17 | 2.23 | [2.01, 2.45] | 17 | 2.06 | [1.77, 2.36] | 21 | 1.48 | [1.38, 1.58] | 13 |
| Teachers don't listen | 1.90 | [1.80, 1.99] | 11 | 2.21 | [1.96, 2.46] | 10 | 2.34 | [2.08, 2.59] | 14 | 2.43 | [2.13, 2.73] | 10 | 1.63 | [1.52, 1.75] | 11 |
| Teachers don't understand | 1.96 | [1.86, 2.05] | 9 | 2.45 | [2.20, 2.70] | 5 | 2.48 | [2.24, 2.72] | 7 | 2.53 | [2.26, 2.80] | 8 | 1.62 | [1.51, 1.74] | 12 |
| Last minute change of plan | 1.81 | [1.72, 1.91] | 13 | 2.34 | [2.08, 2.60] | 6 | 2.45 | [2.21, 2.69] | 8 | 2.67 | [2.39, 2.95] | 5 | 1.40 | [1.29, 1.51] | 16 |
| Not being able to do tasks | 1.64 | [1.55, 1.73] | 18 | 1.97 | [1.72, 2.22] | 16 | 2.10 | [1.85, 2.35] | 20 | 2.00 | [1.69, 2.31] | 24 | 1.39 | [1.29, 1.50] | 17 |
| Being in a chaotic classroom | 1.36 | [1.26, 1.46] | 24 | 1.71 | [1.43, 1.99] | 22 | 2.09 | [1.82, 2.35] | 21 | 2.18 | [1.84, 2.52] | 18 | 0.97 | [0.87, 1.08] | 25 |
| Boring lessons or tasks | 1.62 | [1.53, 1.71] | 19 | 2.13 | [1.90, 2.36] | 12 | 1.96 | [1.71, 2.21] | 24 | 2.14 | [1.87, 2.40] | 20 | 1.34 | [1.23, 1.44] | 18 |
| Being stopped doing enjoyable things | 2.01 | [1.93, 2.10] | 5 | 2.30 | [2.07, 2.53] | 7 | 2.32 | [2.08, 2.56] | 16 | 2.39 | [2.12, 2.67] | 11 | 1.82 | [1.71, 1.92] | 6 |
| Experiencing sensory discomfort | 1.46 | [1.37, 1.55] | 23 | 1.57 | [1.32, 1.82] | 25 | 2.01 | [1.75, 2.27] | 23 | 2.06 | [1.76, 2.36] | 22 | 1.20 | [1.09, 1.30] | 21 |
| Losing and forgetting things | 1.99 | [1.90, 2.09] | 7 | 1.98 | [1.74, 2.22] | 15 | 2.43 | [2.16, 2.71] | 10 | 2.22 | [1.90, 2.53] | 15 | 1.86 | [1.74, 1.98] | 5 |
| In trouble for losing or forgetting | 1.91 | [1.82, 2.00] | 10 | 1.89 | [1.64, 2.14] | 19 | 2.49 | [2.23, 2.75] | 6 | 2.27 | [1.98, 2.55] | 14 | 1.72 | [1.60, 1.84] | 8 |
| Being rushed to complete work | 1.77 | [1.67, 1.86] | 14 | 2.1 | [1.85, 2.35] | 13 | 2.44 | [2.20, 2.68] | 9 | 2.30 | [2.00, 2.60] | 13 | 1.44 | [1.33, 1.55] | 15 |
| Not understanding others | 1.59 | [1.50, 1.68] | 20 | 1.94 | [1.71, 2.17] | 18 | 2.34 | [2.08, 2.59] | 15 | 1.92 | [1.65, 2.20] | 25 | 1.29 | [1.18, 1.40] | 19 |
| Staff treating me unfairly | 2.65 | [2.56, 2.74] | 2 | 2.91 | [2.67, 3.15] | 2 | 3.08 | [2.87, 3.29] | 1 | 3.18 | [2.95, 3.41] | 2 | 2.40 | [2.28, 2.52] | 2 |
| Peers teasing and bullying | 2.17 | [2.07, 2.27] | 3 | 2.20 | [1.91, 2.49] | 11 | 2.59 | [2.34, 2.84] | 3 | 2.77 | [2.49, 3.05] | 3 | 1.96 | [1.84, 2.09] | 3 |
| Being told to try harder | 2.15 | [2.05, 2.24] | 4 | 2.47 | [2.20, 2.74] | 4 | 2.58 | [2.34, 2.82] | 4 | 2.71 | [2.45, 2.97] | 4 | 1.88 | [1.76, 2.00] | 4 |
| Being accused of something I didn’t do | 2.73 | [2.64, 2.82] | 1 | 2.93 | [2.69, 3.17] | 1 | 3.06 | [2.83, 3.28] | 2 | 3.28 | [3.07, 3.49] | 1 | 2.52 | [2.41, 2.64] | 1 |
| Not doing something quite right | 1.97 | [1.88, 2.06] | 8 | 2.53 | [2.29, 2.77] | 3 | 2.39 | [2.17, 2.62] | 13 | 2.61 | [2.33, 2.89] | 6 | 1.64 | [1.53, 1.75] | 10 |
| Not allowed to do self-regulation strategies | 1.71 | [1.61, 1.81] | 16 | 2.23 | [1.96, 2.50] | 9 | 2.41 | [2.17, 2.66] | 12 | 2.58 | [2.31, 2.86] | 7 | 1.28 | [1.17, 1.40] | 20 |
| Being pressured to do well | 2.01 | [1.92, 2.11] | 6 | 2.24 | [1.97, 2.51] | 8 | 2.51 | [2.25, 2.77] | 5 | 2.49 | [2.20, 2.79] | 9 | 1.76 | [1.65, 1.88] | 7 |
| Having too many options | 1.35 | [1.26, 1.44] | 25 | 1.65 | [1.40, 1.90] | 23 | 1.78 | [1.54, 2.02] | 25 | 2.06 | [1.77, 2.36] | 23 | 1.06 | [0.95, 1.16] | 24 |
| Being rushed to move on | 1.58 | [1.49, 1.67] | 21 | 2.09 | [1.85, 2.33] | 14 | 2.22 | [1.99, 2.45] | 18 | 2.32 | [2.03, 2.60] | 12 | 1.19 | [1.10, 1.29] | 22 |
| (c) CUE FREQUENCY × INTENSITY | | | | | | | | | | | | | | | |
| Peers talking behind my back | 2.92 | [2.67, 3.17] | 24 | 2.96 | [2.24, 3.68] | 25 | 4.23 | [3.4, 5.06] | 23 | 4.49 | [3.42, 5.57] | 25 | 2.34 | [2.08, 2.59] | 20 |
| Unexpected wait in a queue | 3.53 | [3.25, 3.82] | 16 | 4.60 | [3.75, 5.45] | 19 | 4.94 | [4.08, 5.81] | 14 | 5.78 | [4.63, 6.94] | 15 | 2.58 | [2.30, 2.86] | 13 |
| Teachers tell me off | 2.83 | [2.59, 3.08] | 25 | 4.04 | [3.17, 4.91] | 21 | 3.59 | [2.94, 4.23] | 25 | 4.75 | [3.75, 5.74] | 24 | 2.06 | [1.82, 2.30] | 24 |
| Schoolmates ignore me | 3.34 | [3.07, 3.61] | 19 | 3.94 | [3.15, 4.73] | 22 | 4.92 | [4.10, 5.75] | 17 | 5.05 | [3.96, 6.15] | 22 | 2.54 | [2.26, 2.82] | 14 |
| Teachers don't listen | 3.36 | [3.04, 3.67] | 18 | 5.38 | [4.42, 6.34] | 14 | 4.62 | [3.64, 5.61] | 20 | 6.00 | [4.83, 7.17] | 13 | 2.16 | [1.85, 2.46] | 22 |
| Teachers don't understand | 3.83 | [3.49, 4.17] | 10 | 6.49 | [5.43, 7.55] | 5 | 5.81 | [4.81, 6.81] | 7 | 6.59 | [5.35, 7.84] | 8 | 2.30 | [1.97, 2.62] | 21 |
| Last minute change of plan | 3.79 | [3.50, 4.09] | 12 | 5.62 | [4.77, 6.47] | 11 | 5.71 | [4.83, 6.60] | 9 | 6.75 | [5.66, 7.84] | 6 | 2.43 | [2.15, 2.71] | 18 |
| Not being able to do tasks | 3.59 | [3.30, 3.88] | 15 | 4.96 | [4.04, 5.88] | 16 | 4.94 | [4.07, 5.81] | 15 | 5.46 | [4.38, 6.54] | 19 | 2.65 | [2.35, 2.95] | 11 |
| Being in a chaotic classroom | 4.03 | [3.68, 4.38] | 8 | 5.44 | [4.43, 6.45] | 13 | 6.39 | [5.36, 7.43] | 2 | 7.32 | [6.01, 8.62] | 3 | 2.60 | [2.26, 2.94] | 12 |
| Boring lessons or tasks | 4.59 | [4.28, 4.91] | 4 | 6.55 | [5.64, 7.46] | 3 | 5.72 | [4.75, 6.69] | 8 | 6.30 | [5.31, 7.30] | 12 | 3.60 | [3.25, 3.95] | 4 |
| Being stopped doing enjoyable things | 3.95 | [3.66, 4.24] | 9 | 5.65 | [4.85, 6.45] | 10 | 4.63 | [3.77, 5.50] | 19 | 5.77 | [4.68, 6.86] | 16 | 3.10 | [2.78, 3.41] | 5 |
| Experiencing sensory discomfort | 3.16 | [2.85, 3.47] | 23 | 3.68 | [2.82, 4.54] | 24 | 5.23 | [4.23, 6.23] | 11 | 5.56 | [4.34, 6.77] | 18 | 2.15 | [1.84, 2.45] | 23 |
| Losing and forgetting things | 3.70 | [3.42, 3.97] | 13 | 4.76 | [3.94, 5.58] | 17 | 4.56 | [3.78, 5.34] | 21 | 5.15 | [4.15, 6.15] | 21 | 3.01 | [2.70, 3.32] | 9 |
| In trouble for losing or forgetting | 3.18 | [2.91, 3.44] | 22 | 4.26 | [3.41, 5.11] | 20 | 3.99 | [3.24, 4.74] | 24 | 4.96 | [3.93, 5.99] | 23 | 2.44 | [2.16, 2.72] | 17 |
| Being rushed to complete work | 4.21 | [3.90, 4.52] | 6 | 5.54 | [4.68, 6.40] | 12 | 6.11 | [5.29, 6.92] | 4 | 6.48 | [5.32, 7.64] | 10 | 3.08 | [2.75, 3.41] | 7 |
| Not understanding others | 3.81 | [3.51, 4.12] | 11 | 5.07 | [4.17, 5.97] | 15 | 6.22 | [5.29, 7.15] | 3 | 5.30 | [4.27, 6.33] | 20 | 2.72 | [2.40, 3.04] | 10 |
| Staff treating me unfairly | 4.24 | [3.90, 4.58] | 5 | 6.80 | [5.73, 7.87] | 2 | 5.19 | [4.24, 6.15] | 12 | 6.67 | [5.42, 7.92] | 7 | 3.04 | [2.69, 3.38] | 8 |
| Peers teasing and bullying | 3.31 | [3.01, 3.62] | 20 | 3.80 | [2.91, 4.69] | 23 | 4.49 | [3.70, 5.28] | 22 | 5.84 | [4.51, 7.16] | 14 | 2.49 | [2.16, 2.83] | 16 |
| Being told to try harder | 4.15 | [3.83, 4.48] | 7 | 5.92 | [4.95, 6.89] | 7 | 4.94 | [4.06, 5.83] | 16 | 6.91 | [5.76, 8.06] | 5 | 3.10 | [2.74, 3.45] | 6 |
| Being accused of something I didn’t do | 5.15 | [4.81, 5.49] | 2 | 7.61 | [6.57, 8.65] | 1 | 5.86 | [4.90, 6.81] | 6 | 7.75 | [6.58, 8.91] | 2 | 3.98 | [3.62, 4.35] | 2 |
| Not doing something quite right | 4.78 | [4.46, 5.10] | 3 | 6.55 | [5.69, 7.41] | 4 | 6.10 | [5.17, 7.02] | 5 | 7.22 | [6.08, 8.35] | 4 | 3.65 | [3.30, 4.01] | 3 |
| Not allowed to do self-regulation strategies | 3.28 | [2.96, 3.59] | 21 | 5.84 | [4.87, 6.81] | 8 | 5.12 | [4.19, 6.06] | 13 | 6.32 | [5.13, 7.50] | 11 | 1.75 | [1.48, 2.03] | 25 |
| Being pressured to do well | 5.86 | [5.49, 6.23] | 1 | 6.48 | [5.43, 7.53] | 6 | 7.79 | [6.63, 8.95] | 1 | 7.77 | [6.50, 9.04] | 1 | 4.94 | [4.52, 5.37] | 1 |
| Having too many options | 3.46 | [3.15, 3.77] | 17 | 4.70 | [3.82, 5.58] | 18 | 4.72 | [3.83, 5.61] | 18 | 5.71 | [4.66, 6.76] | 17 | 2.50 | [2.16, 2.85] | 15 |
| Being rushed to move on | 3.70 | [3.41, 3.99] | 14 | 5.70 | [4.82, 6.58] | 9 | 5.46 | [4.67, 6.26] | 10 | 6.54 | [5.41, 7.68] | 9 | 2.35 | [2.07, 2.63] | 19 |

Note. The mean and 95% confidence interval of the frequency, intensity and frequency x intensity are presented for each MESI CUE across groups. Rank was generated based on the mean values, where the top (smallest number) ranks represent higher frequency, intensity, or the product of frequency x intensity.

## Scale Development

The scree and parallel analysis plots strongly suggested a one-factor structure for the EB latent factor (Fig S5). There was a potential emergence of a second factor close to the elbow of both scree plots.

| **Fig S5. Scree and Parallel Analysis Plots** | |
| --- | --- |
| (a) | (b) |
| 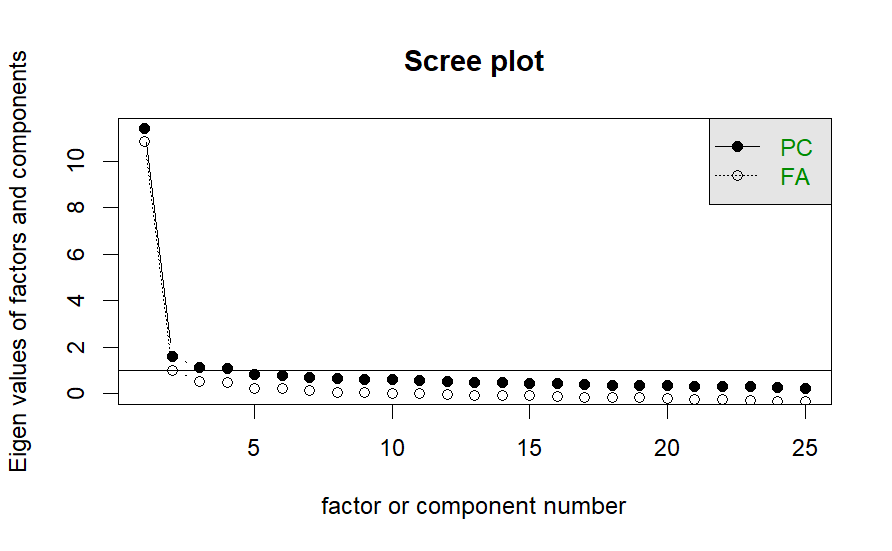 | 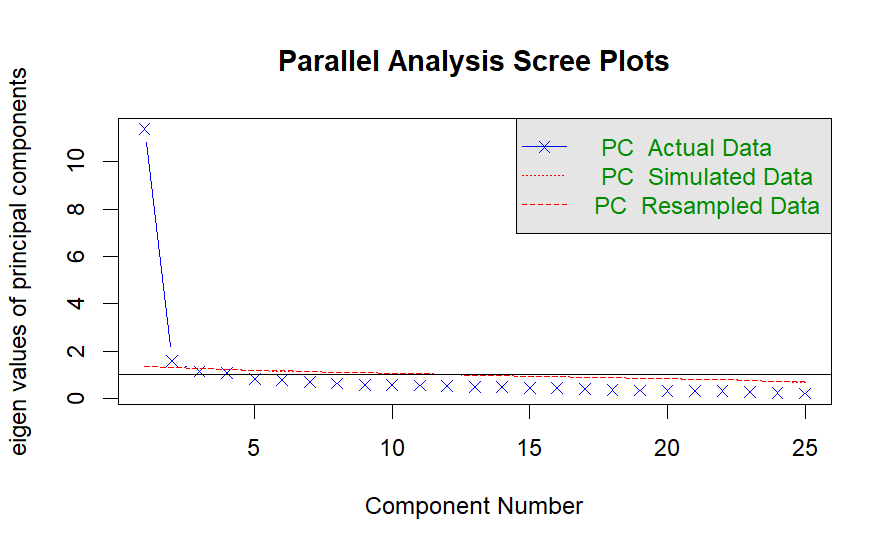 |
| Note. Eigenvalues or variance explained by each principal component (PC) from the principal component analysis and factor (FA) from the factor analysis are plotted in the scree plot (a). Only eigenvalues from the PC of actual data (blue) are plotted together in a parallel analysis plot (b), presented with plots of random eigenvalues or a simulated data line (red). | |

The EFA with a two-factor solution showed that the one CUE *“School staff treating you unfairly”* drove the second factor as this indicator loaded on it with loadings = .92, much higher than other factor loadings in this factor (Table S5a). To investigate this further, we repeated the EFA using principal axis factoring (PAF), which is more robust against skewed data than the OLS, and we produced similar observations (Table S5b).

**Table S5. Loading of Indicators in the Initial EFA with Two-Factor Solution**

|  | 1. OLS | | 1. PAF | |
| --- | --- | --- | --- | --- |
|  | MR1 | MR2 | PA1 | PA2 |
| 1. Peers talking behind my back | **0.70** | -0.07 | **0.70** | -0.07 |
| 1. Unexpected wait in a queue | 0.38 | 0.20 | 0.38 | 0.20 |
| 1. Teachers tell me off | **0.40** | 0.34 | **0.40** | 0.34 |
| 1. Schoolmates ignore me | **0.81** | -0.13 | **0.81** | -0.13 |
| 1. Teachers don't listen | 0.22 | **0.62** | 0.22 | **0.62** |
| 1. Teachers don't understand | 0.37 | **0.53** | 0.36 | **0.53** |
| 1. Last minute change of plan | **0.52** | 0.20 | **0.52** | 0.20 |
| 1. Not being able to do tasks | **0.58** | 0.10 | **0.58** | 0.10 |
| 1. Being in a chaotic classroom | **0.79** | -0.10 | **0.79** | -0.10 |
| 1. Boring lessons or tasks | 0.32 | **0.40** | 0.31 | **0.40** |
| 1. Being stopped doing enjoyable things | 0.19 | **0.57** | 0.19 | **0.57** |
| 1. Experiencing sensory discomfort | **0.66** | 0.03 | **0.66** | 0.03 |
| 1. Losing and forgetting things | **0.54** | 0.07 | **0.54** | 0.07 |
| 1. In trouble for losing or forgetting | **0.46** | 0.22 | **0.46** | 0.22 |
| 1. Being rushed to complete work | **0.59** | 0.19 | **0.59** | 0.19 |
| 1. Not understanding others | **0.78** | -0.04 | **0.78** | -0.04 |
| 1. **Staff treating me unfairly** | -0.14 | **0.92** | -0.14 | **0.92** |
| 1. Peers teasing and bullying | **0.64** | -0.05 | **0.64** | -0.05 |
| 1. Being told to try harder | **0.54** | 0.25 | **0.54** | 0.25 |
| 1. Being accused of something I didn’t do | 0.19 | **0.60** | 0.18 | **0.60** |
| 1. Not doing something quite right | **0.65** | 0.07 | **0.65** | 0.07 |
| 1. Not allowed to do self-regulation strategies | **0.42** | 0.39 | **0.42** | 0.39 |
| 1. Being pressured to do well | **0.59** | 0.04 | **0.59** | 0.04 |
| 1. Having too many options | **0.66** | -0.02 | **0.66** | -0.02 |
| 1. Being rushed to move on | **0.66** | 0.11 | **0.65** | 0.11 |
| Note. Factor loadings of the EB indicators during EFA when a two-factor solution was assumed when the EFA was conducted using ordinary least square (OLS) and using principal axis factoring (PAF) showed high degree of similarity (factor loadings ≥ 0.4 are bold printed). Both methods also showed the item *“Staff treating me unfairly”* drove the emergence of the second factor. Abbreviation. MR = factor loadings based on minimum residuals, PA=factor loadings based on principal axis factoring. | | | | |

Table S6 shows the Cronbach’s alpha and item-total correlation analyses. The overall consistency of the final EB index was Cronbach’s α=0.948. Removing the EB indicator one at a time (with replacement) reduces Cronbach’s α to a value below 0.948 (Raw *α*; Table S6). The reliability of the EB index worsened when each indicator was excluded, indicating the importance of each indicator in the make-up of the EB index. Thus, no indicators were removed based on this observation. The table also showed the average inter-item correlation between each EB indicator with the others. The value is within an ideal range (i.e., *r*=0.15-0.50) indicating that each indicator was well-related to others without any one indicator being redundant. The item-total correlation (raw *r*) values were around the acceptable range (i.e., *r*= 0.30-0.70), although a few indicators, EB6 in particular, had higher raw *r* of 0.78 (reduced to 0.75 when the EB6 is omitted from the computation of total score [see *r* drop])

**Table S6. Cronbach’s Alpha and Item-Total Correlation**

| EB indicator | Raw *α* | Average *r* | Raw *r* | *r* drop |
| --- | --- | --- | --- | --- |
| Peers talking behind my back | 0.946 | 0.436 | 0.64 | 0.61 |
| Unexpected wait in a queue | 0.947 | 0.442 | 0.56 | 0.52 |
| Teachers tell me off | 0.946 | 0.435 | 0.67 | 0.64 |
| Schoolmates ignore me | 0.946 | 0.433 | 0.70 | 0.67 |
| Teachers don't listen | 0.946 | 0.433 | 0.70 | 0.67 |
| Teachers don't understand | 0.944 | 0.428 | 0.78 | 0.75 |
| Last minute change of plan | 0.946 | 0.434 | 0.69 | 0.65 |
| Not being able to do tasks | 0.946 | 0.435 | 0.67 | 0.63 |
| Being in a chaotic classroom | 0.946 | 0.433 | 0.70 | 0.66 |
| Boring lessons or tasks | 0.946 | 0.437 | 0.64 | 0.60 |
| Being stopped doing enjoyable things | 0.946 | 0.437 | 0.64 | 0.61 |
| Experiencing sensory discomfort | 0.946 | 0.435 | 0.68 | 0.64 |
| Losing and forgetting things | 0.947 | 0.439 | 0.60 | 0.57 |
| In trouble for losing or forgetting | 0.946 | 0.437 | 0.64 | 0.60 |
| Being rushed to complete work | 0.945 | 0.431 | 0.74 | 0.71 |
| Not understanding others | 0.945 | 0.431 | 0.74 | 0.71 |
| Peers teasing and bullying | 0.947 | 0.439 | 0.61 | 0.57 |
| Being told to try harder | 0.945 | 0.430 | 0.74 | 0.71 |
| Being accused of something I didn’t do | 0.946 | 0.436 | 0.66 | 0.62 |
| Not doing something quite right | 0.945 | 0.433 | 0.71 | 0.67 |
| Not allowed to do self-regulation strategies | 0.945 | 0.432 | 0.72 | 0.69 |
| Being pressured to do well | 0.947 | 0.438 | 0.63 | 0.58 |
| Having too many options | 0.946 | 0.436 | 0.66 | 0.62 |
| Being rushed to move on | 0.945 | 0.431 | 0.73 | 0.70 |
| Note. Raw *α* = the value of internal consistency measure Cronbach’s *α* when one EB indicator is omitted; average r = average inter-item correlation between each EB indicator with all others; raw r = the item-total correlation, i.e., the correlation between each indicator, with the total EB index including all indicators; r drop = the item-total correlation between each indicator with EB index omitting the value of that indicator in the computation of EB index. | | | | |

## Measurement Equivalence

Measurement invariance across the Groups according to ADHD and/or autism traits, sex, and race (i.e., white vs. non-white) was investigated for the best-fitting EB model involving 24 items is presented in Table S7. We reconfigured ethnicity to race in this investigation instead of the original subgrouping (i.e., White, Black British, Asian British, Mixed Race, and Other ethnic groups) which would result in a relatively small number of participants in the CFA per ethnic subgroups. The final EB model explained 44% of the variance in the data with the model fit (CFI=0.844, TLI = 0.829; RMSEA = 0.089, SRMR = 0.051, 90%CI [0.085, 0.093]; and BIC = 59.5). Taking account of diagnostic groups, sex and race grouping resulted in a reduced model fit in general, but the differences in CFI and RMSEA were minor. The reduced AIC during metric invariance modelling was favourable, suggesting that factor loadings contribute to the latent EB construct to a somewhat similar degree across groups, although the X^2^ model fit remained (unfavourably) significant in some cases. The AIC increased during scalar invariance model fitting, but the degree of invariance at this stringent level for this measure was not expected.

**Table S7. Measurement Invariance of the EB Index Across Various Subgrouping**

| Invariance model | Estimated parameters | *X*^2^ | *df* | *p* | AIC | CFI | RMSEA | SRMR | ΔCFI | ΔRMSEA |
| --- | --- | --- | --- | --- | --- | --- | --- | --- | --- | --- |
| 1. Groups | | | | | | | | | | |
| Configural | 288 | 2981.8 | 1008 | <0.0001 | 91253 | 0.768 | 0.103 | 0.068 | -- | -- |
| Metric | 288 | 3079.24 | 1077 | 0.013 | 91212 | 0.765 | 0.101 | 0.082 | -0.003 | -0.002 |
| Scalar | 291 | 3281.34 | 1146 | <0.0001 | 91276 | 0.749 | 0.101 | 0.090 | -0.016 | <-0.001 |
| 1. Sex | | | | | | | | | | |
| Configural | 144 | 2162.0 | 504 | <0.0001 | 92076 | 0.822 | 0.095 | 0.057 | -- | -- |
| Metric | 144 | 2194.3 | 527 | 0.094 | 92062 | 0.821 | 0.093 | 0.065 | -0.001 | -0.002 |
| Scalar | 145 | 2332.1 | 550 | <0.0001 | 92154 | 0.808 | 0.094 | 0.069 | -0.013 | 0.001 |
| 1. Race (white vs. non-white) | | | | | | | | | | |
| Configural | 144 | 2132.6 | 504 | <0.0001 | 92403 | 0.829 | 0.094 | 0.056 | -- | -- |
| Metric | 144 | 2158.9 | 527 | 0.289 | 92383 | 0.829 | 0.092 | 0.062 | -0.000 | -0.002 |
| Scalar | 145 | 2226.2 | 550 | <0.0001 | 92405 | 0.824 | 0.091 | 0.064 | -0.005 | -0.001 |
| Note. Measurement invariance was investigated across (a) Groups (ADHD, autism, ADHD+autism, neurotypical), (b) sex (c), and (d) race. Model fit metrics were observed in the configural, metric, and scalar invariance models. Abbreviation. X2=chi square statistics, df = degrees of freedom, p= significance. AIC=Akaike Information Criterion, CFI= Comparative Fit Index, RMSEA=Root Mean Square Error of Approximation, SRMR=Standardized Root Mean Square Residual. | | | | | | | | | | |

## Differences of EB Index Across the Groups: Sensitivity Analyses

The EB Index total score across Groups (ADHD, autism, ADHD+autism, neurotypical), by Group*Sex, and by Group*Ethnicity are presented in Table S8. A 2(Sex)*4(Group) ANOVA on the EB index revealed significant main effects of sex (*F*(1,726))=38.9; *p*<.00001), and Group (*F*(3,726)=64.3; *p*<.00001) but not a significant interaction between Sex*Group (*F*(3,726)=0.51; *p*=0.68). Female participants scored higher EB Index than male participants across all diagnostic groups. *Post-hoc* pairwise comparison with TukeyHSD test showed that ADHD and/or autistic participants scored significantly higher EB Index than the neurotypical group (*p*s<.001) after adjusting for differences in sex across groups. Adjusting for sex also revealed higher EB in the ADHD+autism relative to the ADHD group (*p*=.044).

In contrast, a separate 2(Race - white vs. non-white)*4(Group) ANOVA of the EB index revealed a significant main effect of Group only (*F*(3,726)=56.1; *p*<.00001) but neither a main effect of race (*F*(1,726)=0.89; *p*<.35) nor an interaction between these variables (*F*(3,726)=1.05; *p*=0.37), i.e., non-white participants do not differ from white participants in their EB Index ratings and ADHD and/or autistic participants scored significantly higher EB Index than the neurotypical group (*p*s<.001).

**Table S8. EB Index by Group*Sex and by Group*Race**

|  | ADHD  (n=100) | autism  (n=104) | ADHD+autism  (n=79) | neurotypical  (n=452) |
| --- | --- | --- | --- | --- |
| EB Index | 125.5 (66.9) | 126.1 (68.8) | 145.8 (78.0) | 66.5 (52.7) |
| EB Index by Group*Sex |  |  |  |  |
| Female | 154.8 (71.1) | 142.1 (63.8) | 168.4 (83.0) | 84.8 (55.1) |
| Male | 113.0 (61.3) | 106.7 (70.2) | 131.2 (71.7) | 57.82 (49.4) |
| EB Index by Group*Ethnicity |  |  |  |  |
| Asian/Asian British | 43.0 (--) | 90.5 (100.4) | 91.3 (61.8) | 53.9 (38.9) |
| Black/African/Caribbean | 135.0 (65.3) | 134.6 (93.9) | 81.2 (66.4) | 77.2 (59.9) |
| Mixed | 101.1 (63.9) | 139.6 (63.6) | 160.6 (75.6) | 80.9 (62.2) |
| Other ethnic group | 116.5 (12.0) | 172.0 (--) | 229.0 (--) | 66.9 (39.5) |
| Non-white (total of the above) | 110.2 (62.1) | 132.7 (82.0) | 136.2 (79.1) | 66.2 (51.4) |
| White | 130.1 (67.9) | 122.4 (60.6) | 149.5 (77.9) | 67.0 (54.6) |

## Associations between EB Index, ERD, and Mental Health Measures: Sensitivity Analyses

Table S9 lists the findings from the univariate and the multivariate regression models. In addition to the significant univariate associations between increased autistic and ADHD traits, EB, ERD, alexithymia and depression or anxiety, being female was also significantly associated with increased depression and anxiety (*b*_DEP_=0.54, 95% CI [0.39, 0.69]; *b*_ANX_=0.58, 95% CI [0.43, 0.72]), while being non-white was significantly associated with reduction of this mental health problems (*b*_DEP_=-0.18, 95% CI [-0.04, -0.33]; *b*_ANX_=0.66, 95% CI [0.59, 0.70]) (Table S8a).

The multivariate regression models, however, indicated that increased EB, ERD, and alexithymia, but not traits of ADHD or autism, predicted mental health problems (Table S9b), this pattern of association remained after adjusting for sex and race (white vs. non-white) differences across groups (Table S9c-d). Furthermore, race was not predictive of mental health problems in the multivariate model.

**Table S9. Univariate and Multivariate Regression Models Before and After Adjusting for Sex and Race**

|  | Depression (PHQ8) | | | Anxiety (GAD) | | |
| --- | --- | --- | --- | --- | --- | --- |
|  | *b* | *95% CI* | *p* | *b* | *95% CI* | *p* |
| 1. **Univariate** | | | | | | |
| Autistic trait | 0.258 | [0.187, 0.328] | <.001*** | 0.283 | [0.213, 0.352] | <.001*** |
| ADHD trait | 0.371 | [0.303, 0.438] | <.001*** | 0.404 | [0.337, 0.470] | <.001*** |
| EB | 0.601 | [0.543, 0.659] | <.001*** | 0.649 | [0.593, 0.704] | <.001*** |
| ERD | 0.688 | [0.634, 0.741] | <.001*** | 0.698 | [0.645, 0.750] | <.001*** |
| Alexithymia | 0.622 | [0.565, 0.679] | <.001*** | 0.636 | [0.580, 0.692] | <.001*** |
| Sex | 0.540 | [0.394, 0.687] | <.001*** | 0.578 | [0.432, 0.723] | <.001*** |
| Race | -0.190 | [-0.335, -0.045] | .011* | -0.296 | [0.440, 0.152] | <.001*** |
| 1. **Multivariate Baseline Model** | | | | | | |
| Autistic trait | -0.031 | [-0.091, 0.028] | 0.303 | -0.020 | [-0.077, 0.037] | 0.487 |
| ADHD trait | -0.036 | [-0.103, 0.031] | 0.288 | -0.022 | [-0.086, 0.041] | 0.489 |
| EB | 0.290 | [0.218, 0.361] | <.001*** | 0.339 | [0.271, 0.407] | <.001*** |
| ERD | 0.340 | [0.313, 0.487] | <.001*** | 0.365 | [0.282, 0.448] | <.001*** |
| Alexithymia | 0.172 | [0.091, 0.253] | <.001*** | 0.175 | [0.098, 0.252] | <.001*** |
| 1. **Multivariate Model 1 (Adjusting for Sex)** | | | | | | |
| Autistic trait | -0.027 | [-0.087, 0.032] | .366 | -0.016 | [-0.072, 0.041] | .588 |
| ADHD trait | -0.024 | [-0.091, 0.043] | .481 | -0.009 | [-0.073, 0.054] | .772 |
| EB | 0.267 | [0.194, 0.340] | <.001*** | 0.315 | [0.245, 0.384] | <.001*** |
| ERD | 0.398 | [0.311, 0.485] | <.001*** | 0.363 | [0.281, 0.446] | <.001*** |
| Alexithymia | 0.164 | [0.083, 0.245] | <.001*** | 0.165 | [0.089, 0.242] | <.001*** |
| Sex | -0.149 | [-0.260, -0.038] | .008 | -0.163 | [-0.269 -0.058] | .002 |
| 1. **Multivariate Model 2 (Adjusting for Race)** | | | | | | |
| Autistic trait | -0.034 | [-0.094, 0.026] | .262 | -0.018 | [-0.076, 0.039] | .528 |
| ADHD trait | -0.027 | [-0.096, 0.042] | .446 | -0.028 | [-0.094, 0.037] | .400 |
| EB | 0.289 | [0.218, 0.361] | <.001*** | 0.339 | [0.272, 0.407] | <.001*** |
| ERD | 0.402 | [0.314, 0.489] | <.001*** | 0.364 | [0.281, 0.447] | <.001*** |
| Alexithymia | 0.171 | [0.090, 0.252] | <.001*** | 0.175 | [0.099, 0.252] | <.001*** |
| Race | 0.057 | [-0.049, 0.163] | .289 | -0.035 | [-0.136, 0.066] | .493 |

## Additional LDA Results and Sensitivity Analyses

### Additional Four-Group LDA Results

The LDA applied to the four groups (i.e., ADHD, autism, ADHD+autism, and neurotypical) showed lower overall accuracy and grouping reliability than when applied to three groups. The EB indicators could predict the groups with an overall accuracy of 0.68 95% CI [0.65, 0.72] with ‘fair’ grouping reliability (κ =0.28, computed using n-fold cross-validation). The balanced accuracy of the grouping was 0.63 for ADHD, 0.61 for autism, 0.58 for ADHD+autism, and 0.74 for the neurotypical group. The confusion matrices of the predicted versus observed grouping for both the four- and three-group LDA are presented in Fig S6.

**Fig S6. Confusion Matrices of the Predicted vs Observed LDA Grouping**

| (a) | (b) |
| --- | --- |
| 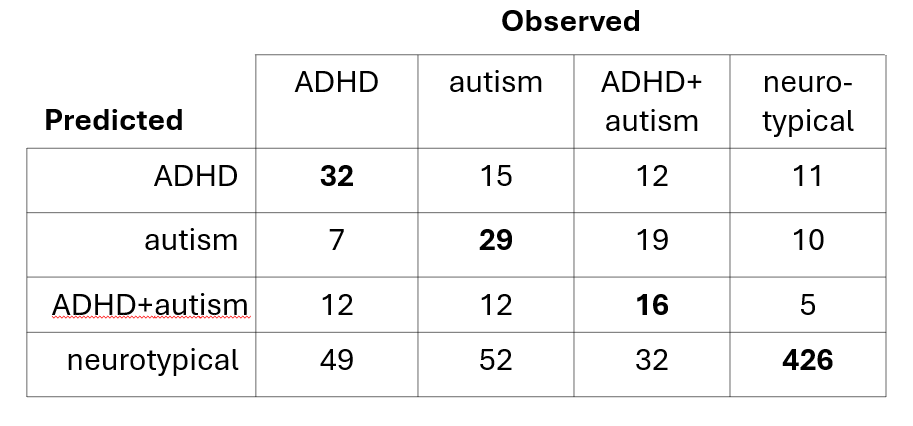 | 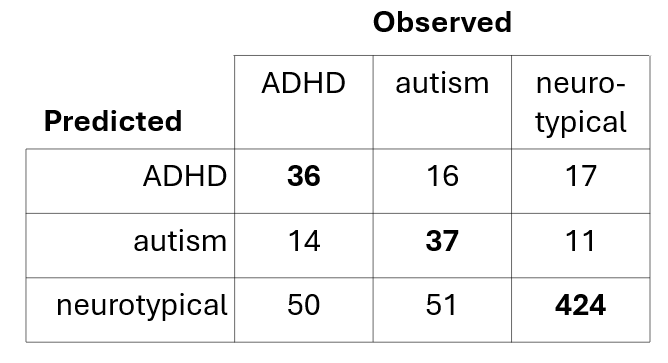 |
| Note. Confusion matrices of the four-group LDA (a) and the three-group LDA (b). The removal of the ADHD+autism group increased the overall accuracy and reliability of the predicted grouping of the LDA, reflected in the increased number of individuals predicted as having ADHD or autism who were diagnosed with the conditions. | |

The LDA model produced three linear discriminant (LD) functions LD1, LD2, and LD3, describing 82.6%, 13.7%, and a negligible proportion of 3.7% variance in the data, respectively (Fig S7a). Similar to the three-group LDA, comparisons of the LD scores in the four-group LDA suggested that the first LD (LD1) differentiated the neurodivergent groups (ADHD, autism, and ADHD+autism) from the neurotypical group (*F*[3,731]=110.2; *p*<.0001; ADHD, autism, ADHD+autism<neurotypical; Δ*M*_neurotypical-ADHD_= 1.41 [95% CI: 1.12, 1.69]; Δ*M*_neurotypical-autism_ =1.24 [95% CI: 0.96, 1.52]; Δ*M*_neurotypical-ADHD+autism_=1.50 [95% CI: 1.18, 1.81], all *ps*<.0001, other comparisons, Fig S7b).

**Fig S7. Four-group LDA Findings**

| 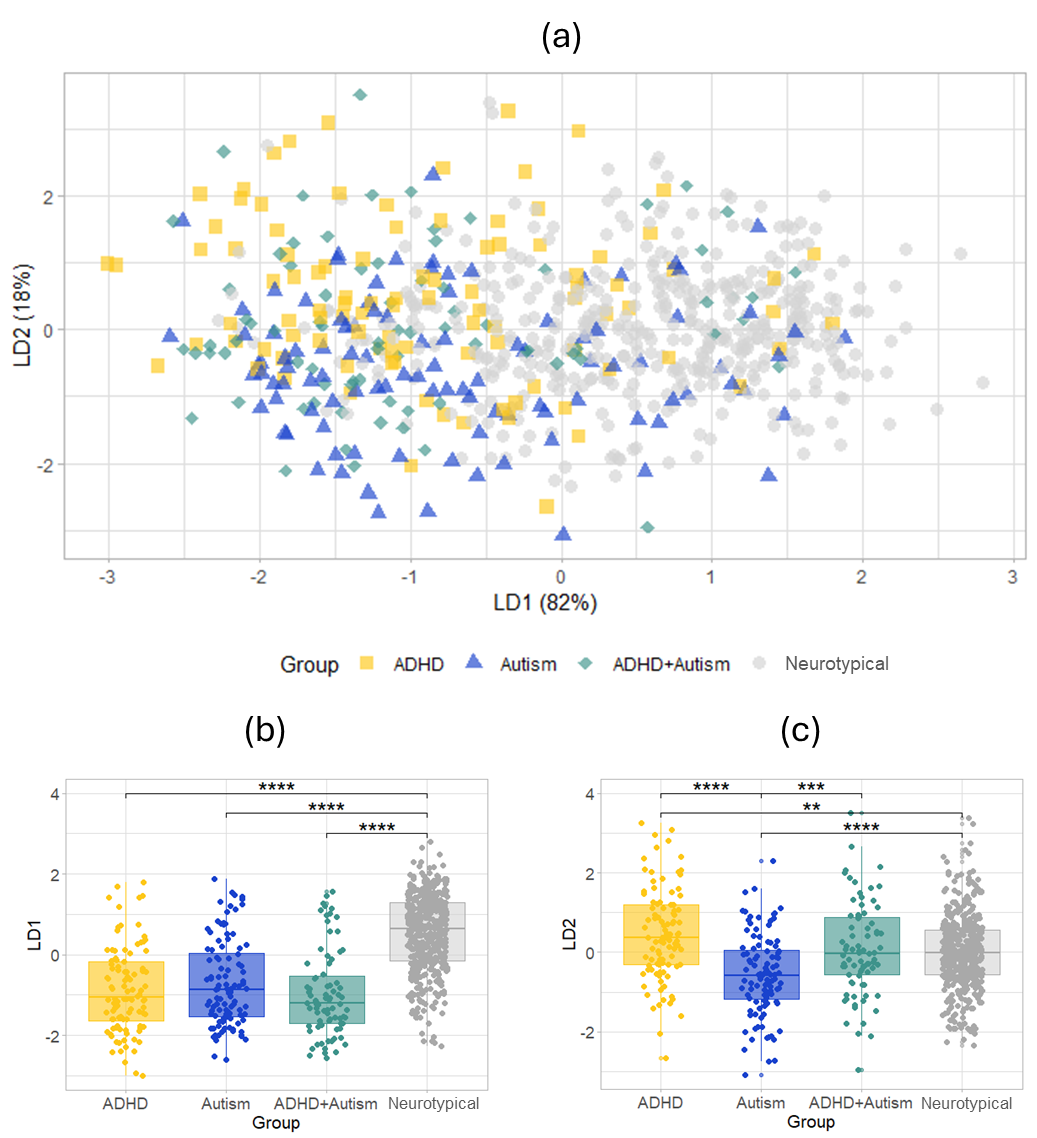 |
| --- |
| Note. The scatterplot of linear discriminants (LD1) and (LD) (a) illustrates the clustering of linear discriminant scores across the predicted diagnostic groups. The neurotypical group tends to cluster on the right side while the neurodivergent groups (ADHD, autism and ADHD+autism) are scattered on the left of the LD1 axis. These were plotted in boxplots with the groups on the x-axis showing how the LD functions help differentiate the groups. ** *p*<.01, *** *p*< .001, **** *p* <.0001 |

The less prominent LD2 appeared to mainly discriminate between the ADHD and the autism group. The significant main effect of Group (*F*[3,731]=18.3; *p*<.0001), was accompanied by a significant pairwise difference between the ADHD and the autism group (ADHD>autism, Δ*M*_ADHD-autism_=1.02 [95% CI: 0.66, 1.38], *p*<.0001), both differed from the neurotypical group (ADHD<neurotypical<autism; Δ*M*_neurotypical-ADHD_=.44 [95% CI: 0.16, 0.72], *p*<.01; Δ*M*_neurotypical-autism_=0.58 [95% CI: 0.30, 0.86]; *p*<.0001). This suggested that the emotional burden experienced by ADHD and autism was determined by distinguishable sets of CUEs. Additionally, the ADHD+autism group was differentiated from the autism group (Δ*M*_ADHD+autism - autism_=0.64 [95% CI: 0.26, 1.03]; *p*<.001), but not from ADHD or neurotypical (Δ*M*_ADHD+autism - ADHD_=-0.37 [95% CI: -0.01, 0.76]; Δ*M*_ADHD+autism – neurotypical_ =0.06 [95% CI: -0.25, 0.38]; both n.s. Fig S7c). The scores of the ADHD+autism group suggested that it is a heterogeneous group, potentially consisting of combined features of the ADHD and the autism alone group. In the LD2 axis, the ADHD+autism group cannot be differentiated in their variation from the neurotypical group. Therefore, to increase the specificity of our findings, we repeated the LDA including only the ADHD, autism, and neurotypical groups, and reported the results in the main manuscript.

### Sensitivity Analyses of the LDA Adjusted for Sex and Race

The 3-group LDA findings, adjusting for sex and race (white vs. non-white) differences across groups, were highly similar to the initial LDA finding. After adjusting for sex, the EB indicators predicted group memberships with an overall accuracy of 0.76 95% CI [0.72, 0.79], while after adjusting for race, the overall accuracy was 0.75 95% CI [0.72, 0.78], both accompanied by a ‘fair’ grouping reliability (κs =0.34 n-fold cross-validations). The balanced accuracies for classifying participants into the ADHD, autism, and neurotypical groups after adjusting for sex (i.e., ADHD [0.66], autism [0.64], and neurotypical [0.71]) and after adjusting for race (i.e., ADHD [0.64], autism [0.64], and neurotypical [0.70]) were fairly similar with the balanced accuracy obtained without the adjustment. The LD functions also explained similar proportions of the variance in the data, 81.7% and 18.3%, after adjusting for sex, and 77.2% and 22.8% after adjusting for race.

Most CUEs that were found specifically more burdensome for the neurodivergent (ADHD, autism) compared to the neurotypical group, and for the ADHD compared to the autism group remained similar to the initial findings (Table S10a) after adjusting for either sex or race (Table S10b-c) , although in some cases the distinction was at a lesser degree (i.e., coefficient values dropped from ≥0.3 to between 0.2 and 0.3). One exception was the CUE *“Peers talking behind my back”*, which was found specifically burdensome for the autism group initially but became non-specific to the diagnostic groups after adjusting for sex differences (Table S10b).

Some of the CUE burdens become more group-specific after adjusting for either sex or race. For instance, “*Teachers don’t understand”*, became more specifically burdensome for the neurodivergent group after adjusting for sex or race (Table S10b-c), and *“Not understanding others”* became more specifically burdensome for the neurodivergent group after adjusting for race alone (Table S10c). *“Peers talking behind my back”*, became more specifically burdensome for the neurotypical group after adjusting for sex or race (Table S10b-c) while *“Experiencing sensory discomfort”* became burdensome for the neurotypical group after adjusting for race only (Table S10c). Lastly, within the neurodivergent subgroups, “*Peers teasing and bullying”* became specifically burdensome for the autism group after adjusting for sex.

**Table S10. Coefficients of the EB Indicators from Each CUE Across the LDA Models**

| MESI CUEs | 1. Initial | | | | 1. Adj. for Sex | | | | 1. Adj. for Race | | | |
| --- | --- | --- | --- | --- | --- | --- | --- | --- | --- | --- | --- | --- |
|  | LD1 | LD1 inf | LD2 | LD2 inf | LD1 | LD1 inf | LD2 | LD2 inf | LD1 | LD1 inf | LD2 | LD2 inf |
| 1.    Peers talking behind my back | 0.261 | neurotypical + | -0.324 | autism ++ | 0.323 | neurotypical ++ | -0.182 | -- | 0.327 | neurotypical ++ | -0.312 | autism ++ |
| 2.     Unexpected wait in a queue | -0.131 | -- | -0.335 | autism ++ | -0.088 | -- | -0.384 | autism ++ | -0.127 | -- | -0.322 | autism ++ |
| 3.     Teachers tell me off | -0.017 | -- | -0.079 | -- | -0.024 | -- | -0.043 | -- | -0.042 | -- | -0.067 | -- |
| 4.     Schoolmates ignore me | 0.054 | -- | -0.115 | -- | 0.043 | -- | -0.17 | -- | 0.112 | -- | -0.117 | -- |
| 5.     Teachers don't listen | -0.103 | -- | 0.309 | ADHD ++ | -0.078 | -- | 0.274 | ADHD + | -0.134 | -- | 0.256 | ADHD + |
| 6.     Teachers don't understand | -0.275 | neurodivergent + | -0.192 | -- | -0.331 | neurodivergent ++ | -0.194 | -- | -0.324 | neurodivergent ++ | -0.146 | -- |
| 7.     Last minute change of plan | -0.393 | neurodivergent ++ | -0.054 | -- | -0.489 | neurodivergent ++ | -0.196 | -- | -0.419 | neurodivergent ++ | -0.123 | -- |
| 8.     Not being able to do tasks | 0.169 | -- | -0.038 | -- | 0.196 | -- | 0.110 | -- | 0.225 | neurotypical + | 0.006 | -- |
| 9.     Being in a chaotic classroom | -0.229 | neurodivergent + | -0.013 | -- | -0.242 | neurodivergent + | 0.115 | -- | -0.281 | neurodivergent + | -0.039 | -- |
| 10.   Boring lessons or tasks | 0.030 | -- | 0.314 | ADHD ++ | 0.012 | -- | 0.228 | ADHD + | 0.027 | -- | 0.263 | ADHD + |
| 11.   Being stopped doing enjoyable things | 0.079 | -- | 0.337 | ADHD ++ | 0.080 | -- | 0.319 | ADHD ++ | 0.074 | -- | 0.332 | ADHD ++ |
| 12.   Experiencing sensory discomfort | 0.257 | neurotypical + | -0.502 | autism ++ | 0.293 | neurotypical + | -0.461 | autism ++ | 0.345 | neurotypical ++ | -0.521 | autism ++ |
| 13.   Losing and forgetting things | 0.063 | -- | 0.328 | ADHD ++ | 0.068 | -- | 0.311 | ADHD ++ | 0.034 | -- | 0.339 | ADHD ++ |
| 14.   In trouble for losing or forgetting | -0.056 | -- | -0.097 | -- | -0.082 | -- | -0.124 | -- | -0.162 | -- | -0.079 | -- |
| 15.   Being rushed to complete work | -0.039 | -- | -0.314 | autism++ | -0.012 | -- | -0.374 | autism ++ | -0.026 | -- | -0.322 | autism ++ |
| 16.   Not understanding others | -0.269 | neurodivergent + | -0.302 | autism ++ | -0.234 | neurodivergent + | -0.324 | autism ++ | -0.310 | neurodivergent ++ | -0.337 | autism ++ |
| 17.   Staff treating me unfairly | -- | -- | -- | -- | -- | -- | -- | -- | -- | -- | -- | -- |
| 18.   Peers teasing and bullying | -0.019 | -- | -0.254 | autism + | -0.067 | -- | -0.389 | autism ++ | 0.078 | -- | -0.223 | autism + |
| 19.   Being told to try harder | 0.150 | -- | 0.422 | ADHD ++ | 0.194 | -- | 0.636 | ADHD ++ | 0.229 | neurotypical + | 0.478 | ADHD ++ |
| 20.   Being accused of something I didn’t do | -0.021 | -- | 0.411 | ADHD ++ | -0.031 | -- | 0.354 | ADHD ++ | -0.107 | -- | 0.367 | ADHD ++ |
| 21.   Not doing something quite right | -0.089 | -- | 0.207 | ADHD + | -0.126 | -- | 0.098 | -- | -0.096 | -- | 0.163 | -- |
| 22.   Not allowed self-regulation strategies | -0.496 | neurodivergent ++ | 0.076 | -- | -0.443 | neurodivergent ++ | 0.158 | -- | -0.466 | neurodivergent ++ | 0.052 | -- |
| 23.   Being pressured to do well | 0.150 | -- | -0.278 | autism + | 0.104 | -- | -0.251 | autism + | 0.092 | -- | -0.282 | autism + |
| 24.   Having too many options | 0.117 | -- | 0.265 | ADHD + | 0.158 | -- | 0.315 | ADHD ++ | 0.242 | neurotypical + | 0.289 | ADHD + |
| 25.   Being rushed to move on | -0.429 | neurodivergent ++ | 0.063 | -- | -0.413 | neurodivergent ++ | 0.031 | -- | -0.393 | neurodivergent ++ | 0.056 | -- |
| Note. Linear discriminant (LD) functions, LD1 and LD2. LD1 distinguished the neurodivergent collectively from the neurotypical group, while LD2 distinguished the ADHD from the autistic group. The absolute value of a coefficient > 0.3 denotes a CUE’s strong influence (++, darker colour cells) on the predicted diagnostic grouping. CUEs with coefficients > 0.2 are also sign-posted (+, lighter colour cells) in the table, indicating lesser but potentially important influences. CUEs indexed neurodivergent (green) exerted influences in the predicted grouping to the neurodivergent group, while CUEs indexed neurotypical (grey) exerted influences in the predicted grouping to the neurotypical group. CUEs indexed autism (blue) or ADHD (yellow) influenced the predicted grouping to the autism or ADHD group, respectively. | | | | | | | | | | | | |

# References

Ajzen, I., & Fishbein, M. (2008). Scaling and Testing Multiplicative Combinations in the Expectancy–Value Model of Attitudes. *Journal of Applied Social Psychology, 38*(9), 2222-2247. doi:10.1111/j.1559-1816.2008.00389.x

Amon, M. J., Annand, C. T., & Holden, J. (2022). Cognitive Dynamics: Additive or Multiplicative? . *Research Directs in Psychology and Behavior, 2*(1). doi:10.53520/rdpb2022.10726

Cattell, R. B. (1966). The Scree Test For The Number Of Factors. *Multivariate Behavioral Research, 1*(2), 245-276. doi:10.1207/s15327906mbr0102_10

de Winter, J. C., Dodou, D., & Wieringa, P. A. (2009). Exploratory Factor Analysis With Small Sample Sizes. *Multivariate Behav Res, 44*(2), 147-181. doi:10.1080/00273170902794206

Dhamnetiya, D., Goel, M. K., Jha, R. P., Shalini, S., & Bhattacharyya, K. (2022). How to Perform Discriminant Analysis in Medical Research? Explained with Illustrations. *J Lab Physicians, 14*(4), 511-520. doi:10.1055/s-0042-1747675

Hastie, T., Tibshirani, R., & Friedman, J. (2009). Model Assessment and Selection. In T. Hastie, R. Tibshirani, & J. Friedman (Eds.), *The Elements of Statistical Learning: Data Mining, Inference, and Prediction* (pp. 219-259). New York, NY: Springer New York.

Horn, J. L. (1965). A rationale and test for the number of factors in factor analysis. *Psychometrika, 30*, 179-185. doi:10.1007/bf02289447

Lambert, Z. V., & Durand, R. M. (1975). Some precautions in using canonical analysis. *Journal of Marketing Research, 12*(000004), 468. doi:10.1177/002224377501200411

Nunnally, J. C. (1978). *Psychometric Theory (2nd ed.)*. New York: McGraw Hill.

Pearson, R., Mundfrom, D. J., & Piccone, A. (2013). A Comparison of Ten Methods for Determining the Number of Factors in Exploratory Factor Analysis. *Multiple Linear Regression Viewpoints, 39*.

Streiner, D. L., & Norman, G. R. (2008). *Health Measurement Scales : A practical guide to their development and use*: Oxford University Press, Incorporated.

Tofallis, C. (2014). Add or Multiply? A Tutorial on Ranking and Choosing with Multiple Criteria. *INFORMS Transactions on education, 14*(3), 109-119. doi:10.2139/ssrn.3762021
